# A specific H_2_/CO_2_ consumption molar ratio of 3 as a signature for the chain elongation of carboxylates from brewer’s spent grain acidogenesis

**DOI:** 10.3389/fbioe.2023.1165197

**Published:** 2023-06-01

**Authors:** Grégoire B. L. Henry, Florent Awedem Wobiwo, Arnaud Isenborghs, Thomas Nicolay, Bruno Godin, Benoit A. Stenuit, Patrick A. Gerin

**Affiliations:** ^1^ Laboratory of Bioengineering and Biorefining, Earth and Life Institute—Applied Microbiology, Université Catholique de Louvain, Louvain-La-Neuve, Belgium; ^2^ Walloon Agricultural Research Center (CRA-W), Valorization of Agricultural Products Department, Gembloux, Belgium

**Keywords:** complex organic feedstock, acidogenic fermentation, methane-arrested anaerobic digestion, medium-chain carboxylates, H_2_/CO_2_ conversion, Wood-Ljungdahl pathway, reverse β-oxidation

## Abstract

Brewer’s spent grain (BSG) is an undervalorized organic feedstock residue composed of fermentable macromolecules, such as proteins, starch, and residual soluble carbohydrates. It also contains at least 50% (as dry weight) of lignocellulose. Methane-arrested anaerobic digestion is one of the promising microbial technologies to valorize such complex organic feedstock into value-added metabolic intermediates, such as ethanol, H_2_, and short-chain carboxylates (SCC). Under specific fermentation conditions, these intermediates can be microbially transformed into medium-chain carboxylates through a chain elongation pathway. Medium-chain carboxylates are of great interest as they can be used as bio-based pesticides, food additives, or components of drug formulations. They can also be easily upgraded by classical organic chemistry into bio-based fuels and chemicals. This study investigates the production potential of medium-chain carboxylates driven by a mixed microbial culture in the presence of BSG as an organic substrate. Because the conversion of complex organic feedstock to medium-chain carboxylates is limited by the electron donor content, we assessed the supplementation of H_2_ in the headspace to improve the chain elongation yield and increase the production of medium-chain carboxylates. The supply of CO_2_ as a carbon source was tested as well. The additions of H_2_ alone, CO_2_ alone, and both H_2_ and CO_2_ were compared. The exogenous supply of H_2_ alone allowed CO_2_ produced during acidogenesis to be consumed and nearly doubled the medium-chain carboxylate production yield. The exogenous supply of CO_2_ alone inhibited the whole fermentation. The supplementation of both H_2_ and CO_2_ allowed a second elongation phase when the organic feedstock was exhausted, which increased the medium-chain carboxylate production by 285% compared to the N_2_ reference condition. Carbon- and electron-equivalent balances, and the stoichiometric ratio of 3 observed for the consumed H_2_/CO_2_, suggest an H_2_- and CO_2_-driven second elongation phase, converting SCC to medium-chain carboxylates without an organic electron donor. The thermodynamic assessment confirmed the feasibility of such elongation.

## 1 Introduction

The current environmental crisis, triggered by the overuse of non-renewable resources and overexploitation of natural ecosystems by an ever-growing population, gave a compelling impetus for the development of bio-based circular economy. Shaping a sustainable society requires an economically viable bio-based chemical industry. Nowadays, scientists and engineers are developing various biorefinery processes to valorize biomass into biofuels and chemicals in order to decrease our dependence on fossil carbon resources (Yaoyang and Boeing, 2013; Obileke et al., 2022). Among all the biomass available, the low-cost organic residues that do not compete with food production, such as wood residues, agricultural and agro-industrial wastes, municipal solid wastes, food wastes, or sewage sludges, have been highlighted as relevant feedstocks to produce a variety of platform molecules. Different conversion processes can be followed depending on the physicochemical properties of the biomass (e.g., chemical composition, moisture content, and heterogeneity) and the final targeted products (Holtzapple et al., 2021). Holtzapple and Granda (2009) compared thermochemical, sugar, and carboxylate biorefinery platforms to valorize lignocellulosic biomass containing polysaccharides (68.3%) and lignin (31.7%). The *in silico* highest product yield in liquid biofuels was obtained for the carboxylate platform. Once carboxylates are microbially obtained from organic feedstocks, they can easily be converted by conventional chemistry into fuels and chemicals (Holtzapple and Granda, 2009; Agler et al., 2011; Albuquerque et al., 2011; Lee et al., 2014; Stamatopoulou et al., 2020; Holtzapple et al., 2021).

The carboxylate platform aims at transforming biomass to carboxylates, thanks to microbial communities. This process is also identified as methane-arrested anaerobic digestion operating like the anaerobic digestion pathway except that the last step of methanogenesis must be completely inhibited to keep a mixture of short-chain carboxylates (SCC), short-chain alcohols (SCA), lactate, and H_2_ as final products (Holtzapple et al., 2021). These molecules have better economic value than methane (Perimenis et al., 2018). In recent years, there has been increasing interest in the transformation of these intermediate compounds into medium-chain carboxylates (MCC) by chain elongation metabolism (CE). MCC are structurally composed of an aliphatic carbon chain of 6–10 carbon atoms, with a carboxylic acid group on a terminal carbon. Due to their longer aliphatic carbon chain, MCC are less water soluble than SCC. Thus, they are easier to recover from the aqueous fermentation broth (Spirito et al., 2014).

The identified CE pathway corresponds to the reverse β-oxidation (RBO) mechanism, that is, the addition to an existing carboxylate of a two-carbon acetyl-CoA derived from ethanol or lactate. The investigation of the RBO pathway with pure cultures and simple substrates allowed for identifying two main conditions required to promote microbial-driven chain elongation: a) the presence of an energy-rich electron donor, such as ethanol (EtOH, six electron-equivalents/mole_C_), lactate (four electron-equivalents/mole_C_), or H_2_ (two electron-equivalents/mole_H2_) as a precursor of acetyl-CoA and b) the maintenance of a reducing environment (Agler et al., 2011; Zhang et al., 2013; Spirito et al., 2014; Angenent et al., 2016; Bengelsdorf et al., 2018; Holtzapple et al., 2021). Usually, laboratory-scale studies opted for the supplementation of pure substrates, such as ethanol, lactate, or glucose, to increase MCC production (Grootscholten et al., 2014; Roghair et al., 2018). Indeed, the use of complex biomass as organic substrates, even with a high degree of reduction, enables limited MCC production rates and yields (Baleeiro et al., 2021). On an industrial scale, a biorefinery process must use low-cost feedstocks and be independent of the expensive supply of electron donors, such as chemical-grade reagents.

Low-cost organic feedstocks available to develop biorefining processes, such as woody or herbaceous biomass, are mainly composed of non-fermentable lignocellulosic compounds (Holtzapple et al., 2021). Conversely, brewer’s spent grain (BSG) is a promising feedstock alternative, with a world production estimated at 36.4 million tons in 2021 (Zeko-Pivač et al., 2022). It comprises at least 50% (as dry weight) of lignocellulose and contains proteins, soluble carbohydrates, and residual starch as fermentable molecules (Mussatto et al., 2006; Mussatto, 2014; Bachmann et al., 2022; Miranda et al., 2022). The use of BSG as an organic feedstock to produce carboxylates could be of great interest from an industrial bioprocess perspective. In order to increase the production of MCC in an integrated bioprocess from BSG, the fermentable fraction could be directly used in a methane-arrested anaerobic digestion process to produce the metabolic intermediates (SCC, alcohols, lactate, H_2_, and CO_2_), whereas the unfermented lignocellulosic fraction would be recovered at the end of the fermentation and valorized, for example, by gasification to generate syngas (mainly CO_2_, CO, and H_2_) and water-gas shifted syngas (CO_2_ and H_2_) (Baleeiro et al., 2019; Giuliano et al., 2020).

To promote CE, CO_2_, CO, and H_2_ gases would be used as a second source of carbon and electron donors through the Wood–Ljungdahl pathway catalyzed by acetogens (Bengelsdorf et al., 2018). This pathway, also called the reductive acetyl-CoA pathway, converts these gases into acetyl-CoA which can be converted into different products such as acetate or ethanol. Like acetate and ethanol, acetyl-CoA is a key metabolite in the CE process. Baleeiro et al. (2019), Baleeiro et al. (2021), and Baleeiro et al. (2022) hypothesized to promote MCC production by combining syngas fermentation with biomass fermentation. Some studies showed that H_2_ positively affects MCC production (Steinbusch et al., 2011; Nzeteu et al., 2018). Nevertheless, only a few studies have focused on the effect of the combination of organic substrate, H_2_, and CO_2_ for medium-chain carboxylate production catalyzed by microbial communities (Arslan et al., 2012; Zhang et al., 2013; Darvekar et al., 2019; González-Tenorio et al., 2020; Baleeiro et al., 2021).

The aim of this study is i) to determine the fermentation profiles and conversion yields that can be obtained from BSG as complex organic feedstock, ii) to compare the titers, rates, and yields of fermentation products when BSG is supplemented with exogenous H_2_ and CO_2_, individually or together, as secondary, inorganic electron and carbon sources, iii) to investigate the role of H_2_ partial pressure on MCC production, and iv) to have a better understanding of the MCC production mechanisms when both organic and inorganic (H_2_ and CO_2_) feedstocks are supplied to microbial ecosystems.

## 2 Materials and methods

### 2.1 Substrate

BSG was used as an organic substrate for microbial fermentation. It was supplied by the Bertinchamps craft brewery in Gembloux, Belgium. BSG was received fresh the day of the brew, directly divided into 1 kg bags, and stored in the freezer at −20°C before use for fermentation. A fraction of the wet BSG was directly dried at 50°C for further chemical composition analyses.

The chemical composition of the initially dried BSG was analyzed. Polysaccharides were quantified by the Van Soest method (Godin et al., 2011), with the addition of thermotolerant alpha-amylase to degrade starch. Hydrolysates were quantified by liquid chromatography using the method described by Godin et al. (2011), which uses a refractive index detector. Lignin content was quantified by the Van Soest gravimetric method described in Godin et al. (2016). The protein content was determined by the Dumas method using 6.25 g_protein_ g^−1^_N_ as the conversion factor of nitrogen to protein. The content of mineral compounds was determined gravimetrically after organic matter oxidation in a muffle furnace set at 550°C up to constant mass. The composition of the initial BSG is presented in Table 1.

**TABLE 1:** Composition of the BSG used for fermentation. FM, fresh matter; TS, total solids; VS, volatile solids; COD, chemical oxygen demand.

Component	Amount
Total solid_105°C_ (g_TS_ g^−1^_FM_)	0.228 ± 0.004
Volatile solid_550°C_ (g_VS_ g^−1^_TS_)	0.9582 ± 0.0004
COD_TS_ (g_COD_ g^−1^_TS_)	1.54 ± 0.01
Lignin (g_lignin_ g^−1^_TS_)	0.048 ± 0.001
Hemicelluloses (g_hemicelluloses_ g^−1^_TS_)	0.331 ± 0.006
Cellulose (g_cellulose_ g^−1^_TS_)	0.178 ± 0.002
Proteins (g_proteins_ g^−1^_TS_)	0.251 ± 0.003
Starch (g_starch_ g^−1^_TS_)	0.069 ± 0.002
Soluble sugars (g_soluble_sugars_ g^−1^_TS_)	0.0077 ± 0.0001
Minerals (g_minerals_ g^−1^_TS_)	0.042 ± 0.001
Other (g_other_ g^−1^_TS_)	0.070 ± 0.015

### 2.2 Inoculum

Return-activated sludge samples from the municipal wastewater treatment plant in Mont-Saint-Guibert, Belgium, were used as primary inoculum for fermentation. The treatment plant combines enhanced biological phosphorus removal and the nitrification–denitrification process. The sludge did not receive any pretreatment or concentration before use. The average properties of sludge were analyzed in triplicate: total solids (TS) in the fresh matter (FM) at 0.0092 ± 0.0002 g_TS_ g^−1^_FM_, volatile solids (VS) at 0.467 ± 0.003 g_VS_ g^−1^_TS_, and chemical oxygen demand (COD) at 7.87 ± 0.49 g_COD_ kg^−1^_FM_.

### 2.3 Reagents

A mineral medium was supplemented to the broth from three concentrated stock solutions (a., b., c.) containing a. NH_4_H_2_PO_4_; b. MgCl_2_.6H_2_O, MgSO_4_.7H_2_O, CaCl_2_.2H_2_O, KCl; and c. H_3_BO_3_, FeCl_2_.4H_2_O, ZnSO_4_, MnCl_2_, CoCl_2_, CuCl_2_, NiCl_2_, Na_2_MoO_4_, Na_2_Se_2_O_3_.

The final concentrations in the bioreactor were NH_4_H_2_PO_4_ = 3.6 g L^−1^, MgCl_2_.6H_2_O = 0.6 g L^−1^, MgSO_4_.7H_2_O = 0.2 g L^−1^, CaCl_2_.2H_2_O = 0.2 g L^−1^, KCl = 0.15 g L^−1^, H_3_BO_3_ = 3 mg L^−1^, FeCl_2_.4H_2_O = 15 mg L^−1^, ZnSO_4_ = 1 mg L^−1^, MnCl_2_ = 0.3 mg L^−1^, CoCl_2_ = 2 mg L^−1^, CuCl_2_ = 0.1 mg L^−1^, NiCl_2_ = 0.2 mg L^−1^, Na_2_MoO_4_ = 0.3 mg L^−1^, and Na_2_Se_2_O_3_ = 0.1 mg L^−1^.

Solutions of 2 M KOH and 1 M H_3_PO_4_ were used to adjust the pH.

### 2.4 Bioreactor preparation and monitoring

**FIGURE 1:**
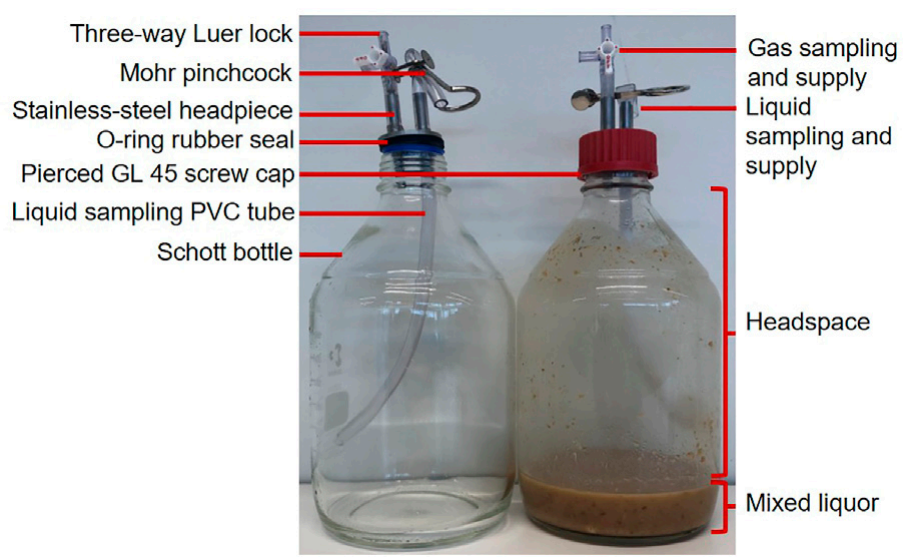
Picture of assembled and filled bioreactors.

Fermentations were performed in 2-L Scott Duran^®^ GL45 glass bottles as a bioreactor, with a mixed liquor working volume of 0.22 L (Figure 1). The GL45 cap was made of a stainless steel headpiece with a two-tube nozzle. The headpiece was pressed on a rubber O-ring seal by a GL45 PBT screw cap with an aperture. Each tube of the headpiece was connected to a flexible PVC tube outside the bottle. The first tube was sealed with a three-way Luer lock valve for gas sampling or supplementation and the second tube with a Mohr pinchcock for liquid sampling or supplementation. The inside part of the tube dedicated to liquid sampling was sealed with a PVC tube long enough to reach the mixed liquor (ML) (Figure 1). The bioreactor was weighed empty and full of water, and the difference between the values allowed for determining the specific headspace volume of each bioreactor.

The ML contained a BSG concentration of 87 g_COD_ L^−1^_ML_ in an initial total volume of 0.22 L. This was achieved by adding 54 g_FM_BSG_, 20 mL of inoculum (7.9 g_COD_ L^−1^), 11 mL of the mineral stock solutions, and 153 mL of water into each bioreactor. The initial ML pH was 5.28 ± 0.12. It was adjusted to pH 7.07 ± 0.03 by adding 3 mL of 2 M KOH to each bioreactor before the beginning of the fermentation. The pH were checked with a pH-meter (WTW pH-Electrode SenTix^®^ 41, WTW Multiline P4 universal meter). Each supply was weighed with a scale (OHAUS navigator NVT6201) and recorded after each addition to the bioreactor. A total of 10 mL of mixed liquor was taken with a 50-mL polypropylene syringe (BD Plastipack, United States) to analyze the ML initial composition. Bioreactors were then sealed and flushed for 5 min at a 4 L min^−1^ flow rate with the appropriate gas depending on the conditions tested (Table 2). Then, 20 mL gaseous samples were taken to analyze the starting headspace gas composition to ensure no trace of remaining O_2_. The gas pressure was measured with a hand manometer (UNIK 5,000 manometer, GE, United States) and adjusted by gas addition to the desired value with the desired gas (Table 2). Bioreactors were incubated in the dark, in a thermostated room at 35°C on an orbital shaker at 120 rpm for 21 days (Kühner Shaker Lab-Shaker).

**TABLE 2:** Headspace gas used for the different tested conditions.

Gas used	Adjusted absolute pressure (bar)	Flushed after monitoring	Origin	Purity
N_2_	1.3	No	Air liquide	99.999%
			P0271 Alphagaz 1	
H_2_	1.3	No	Air liquide	99.999%
			P0231 Alphagaz 1	
H_2_	1.6	No	Air liquide	99.999%
			P0231 Alphagaz 1	
CO_2_	1.3	No	Air liquide	99.7%
			UN-Nr 1013	
H_2_/CO_2_	1.3	Yes^a^	Air liquide	77.13/22.87%_mol_
77/23%_mol_			Mixture Krystal	

^a^ Bioreactors were flushed to keep the H_2_/CO_2_ ratio at the target value of 3.35 and compensate for the differential consumption of the two individual gases. The H_2_/CO_2_ proportion of 77/23%_mol_ has been chosen according to the suggested approximation of De Groof et al. (2019) (1 bar of *p*H_2_ and 0.3 bar of *p*CO_2_) based on the thermodynamic study of Weimer and Kohn (2016) showing an optimal H_2_/CO_2_ ratio of 3–4 for chain elongation.

The bioreactor monitoring routine on each measurement day was performed as illustrated in Supplementary Figure S8. In the thermostated room, for each bioreactor, the initial bioreactor mass was recorded, the initial pressure was measured, an initial gas sample was analyzed, and a liquid sample was collected with a 50-mL syringe (BD Plastipack). Sampling and supply of liquid and gas took place without external air entry or release of gas from the headspace, thanks to the three-way Luer lock and Mohr pinchcock device (Figure 1).

The syringes containing ML were weighed to determine the mass of the ML sample by subtraction of the tare. The remaining ML in the bioreactors was calculated by weight difference. A 2-mL sampled ML was transferred to a glass tube for pH measurement and titration. According to the titration and the remaining bioreactor ML quantities, the adequate volume of 2 M KOH or 1 M H_3_PO_4_ was added to each reinjection syringe for bioreactor pH adjustment to 7.0 (see next paragraph). The remaining ML was centrifuged (Thermo Scientific Heraeus Megafuge 40 centrifuge), and the liquid and solid phases were separated and weighed separately in 15-mL Falcon^®^ tubes. Adequate volumes were collected from the liquid fractions to carry out soluble chemical oxygen demand (COD) measurements and extract organic soluble metabolites. The solid fractions and the remaining liquid fractions were stored at −20°C.

The content of reinjection syringes was introduced into the appropriate bioreactors for pH adjustment. Depending on the condition (Table 2), a flush of the headspace or simple supplementation of the appropriate gas was operated to reach 20 mbar above the desired pressure. A final gas sample was collected to determine the final gas composition and check for the absence of O_2_ in the headspace. Then, the resulting pressure was measured. The variation of composition and pressure between the two monitoring days allowed for calculating the gas consumption/production through the ideal gas law. The bioreactor monitoring routine was finalized by a final mass measurement of each bioreactor to determine the remaining ML mass in the bioreactor.

At the end of the 21 days of fermentation, the remaining ML was weighed by difference with the mass of empty bioreactors. After ML centrifugation, the supernatant and pellet fractions were weighed and analyzed for total solids, volatile solids, ash content, soluble COD content, metabolite composition, and total COD (COD_t_) of the solid fraction.

The mean of the final ML volume on day 21 was 198 ± 19 mL. The differences between bioreactors resulted from the different sampling and neutralization solution volumes taken and added. Therefore, the metabolite mass production was normalized by the actual volume of the ML and expressed as kg_COD_ normalized by the initial volume of the mixed liquor (kg_COD_ m^−3^_ML_).

### 2.5 Analytical method

Total solids (TS), volatile solids (VS), ash content, and COD_t_ were measured according to standard methods (APHA, 1998). Soluble COD was measured using the COD Cell Test method (Spectroquant^®^ kits 1.14555.0001, Spectroquant Pharo 300, Merck KGaA, Germany) according to the provider’s instructions.

The headspace gas composition was analyzed by gas chromatography (CompactGC^3.0^, Global Analyser Solution) with a thermal conductivity detector (GC-TCD). This gas analyzer was calibrated to quantify the proportion of H_2_, N_2_, O_2_, CO_2_, CH_4_, and H_2_O present in the sample. The chromatograph combined two channels. The front channel was equipped with an Rt-QBond column (10 m × 0.32 mm), which separated CO_2_ and H_2_O from the other gases. The back channel included two columns in series: one similar to the front channel (Restek, France, Rt-QBond, 2 m × 0.32 mm), which retained CO_2_, followed by a Molsieve 5A column (7 m × 0.32 mm, Restek, France) that separated the other gases. A three-way valve between the two columns allowed for back-flushing CO_2_ so that it did not enter the Molsieve column. The elution was performed under isotherm conditions at 60°C with helium as carrier gas at 1 mL min^−1^ and a pressure of 80 kPa for the front channel and at 70°C with argon as carrier gas at 1 mL min^−1^ and a pressure of 70 kPa for the back channel. Detectors were heated at 90°C and the filaments at 170°C.

Soluble metabolites were extracted from ML supernatant with liquid–liquid extraction in diethyl ether for GC analysis (Andersen et al., 2017). Extractions were carried out in glass screw top Pyrex test tubes, with approximately 1 g of NaCl (for salting out), 1.5 mL of distilled water, 0.5 mL of the sample or calibrating solution, 0.2 mL of the 4-methyl valerate (4-MV) as internal standard (1 g L^−1^ stock solution). Finally, 1 mL of 2 M H_2_SO_4_, immediately followed by 2 mL of pure diethyl ether (Sigma-Aldrich), was added to each test tube before quickly closing them. The tubes were then manually vigorously shaken for 1 min and centrifuged for 1 min at 3,000 rpm to reach perfect phase separation. Once centrifuged, the organic phase of each test tube was collected with a micropipette and transferred to a glass vial sealed with a PTFE/silicone screw cap (VWR, Germany).

Ether extracts were analyzed by gas chromatography (Trace GC, Thermo Scientific). The samples were injected using an automated sampler. The injector was in a split mode (1:10; split flow 12 mL min^−1^) and maintained at 250°C. N_2_ was used as carrier gas at a constant flow of 1.2 mL min^−1^. Separation was performed by a 30 m, 0.25 mm ID, polar column (DB-WAX Ultra Inert GC Columns, Agilent). Detection was performed using a flame ionization detector (FID) at 260°C. The run time was 43 min. The oven temperature was set at 40°C and increased after 1 min by 10°C min^−1^ for 20 min. It remained at 240°C for 22 min. Chromeleon 7 was used as identification and quantification software. Chromatograms were automatically integrated; each peak area was normalized by the peak area of the 4-MV internal standard and finally converted to concentration using the corresponding calibration factor. Two stock solutions with alternating component concentrations were used to determine the calibration lines. These stock solutions were diluted with three dilution factors (2/3, 4, and 20) and injected to obtain a final calibration line containing nine points for each component [(0; 0) included]. The components detected and quantified were ethanol (EtOH), propanol (PropOH), isopropanol (iPropOH), butanol (ButOH), isobutanol (iButOH), acetate (C2), propionate (C3), butyrate (C4), isobutyrate (iC4); pentanoate (C5), isopentanoate (iC5), hexanoate (C6), heptanoate (C7), octanoate (C8), nonanoate (C9), decanoate (C10), phenylacetate (PhC2), and phenylpropionate (PhC3).

### 2.6 Statistics

The experiment started with three biological replicates for the N_2_, H_2_ 1.3 bar, and H_2_&CO_2_ conditions; only duplicates were available for the CO_2_ and H_2_ 1.6 bar conditions. For the H_2_&CO_2_ condition, one replicate was affected by methanogenesis and excluded. Results are based on the mean with standard deviations for the triplicates and average deviation for duplicates.

## 3 Results

BSG was fermented by a mixed microbial inoculum under N_2_ at 1.3 bar as a reference condition. In the tested conditions, the headspace was fed with a mixture of H_2_ and CO_2_ at 1.3 bar, H_2_ alone at 1.3 and 1.6 bar, and CO_2_ alone at 1.3 bar to investigate the influence of the supplementation of inorganic carbon and reducing equivalent sources on the methane-arrested anaerobic digestion of BSG (Table 2).

### 3.1 Biomass solubilization and fermentation kinetics

**FIGURE 2:**
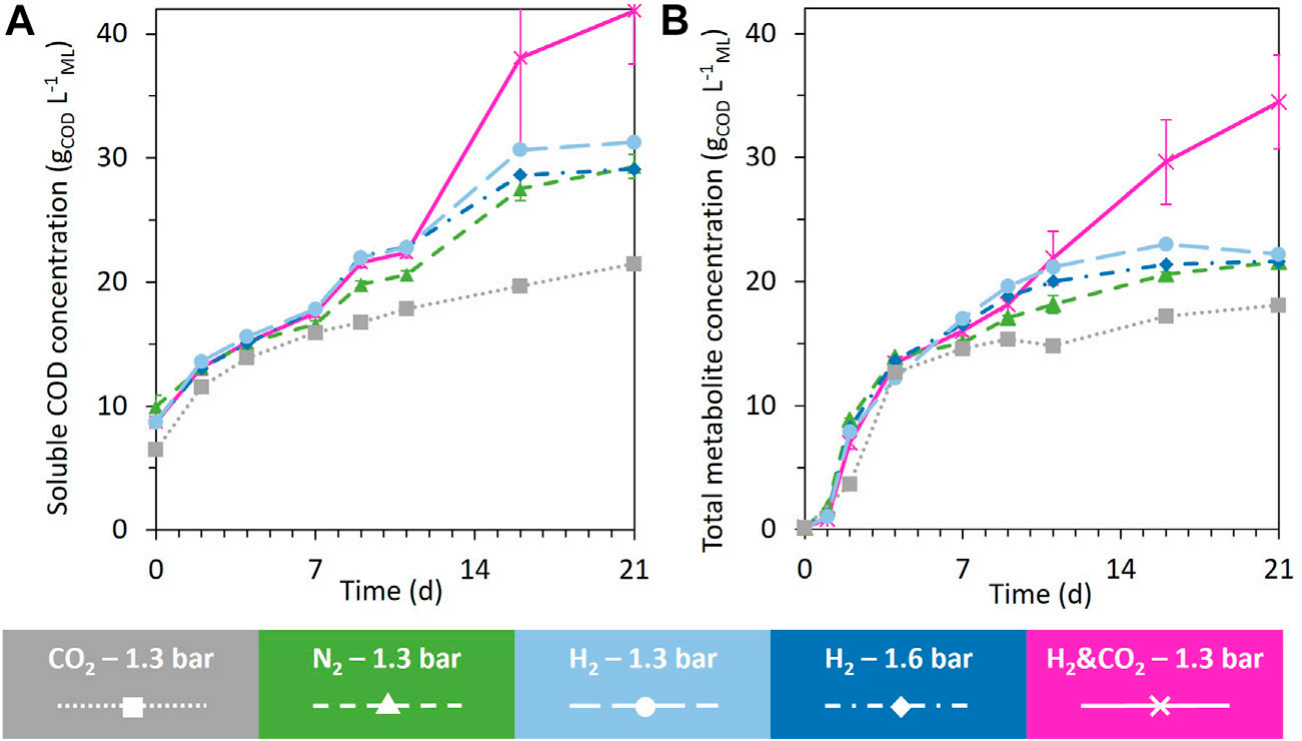
Evolution of the soluble COD concentration **(A)** and the total identified metabolites **(B)** during the 21 days of fermentation under all tested conditions. When error bars are not visible, they are present but smaller than the dot symbol.

Figure 2 presents the evolution of the soluble COD and identified total metabolites over time. Up to day 7, all conditions led to similar total metabolites and soluble COD concentrations. After day 7, the evolution of these concentrations was affected by the headspace composition (Figure 2B). At the end of the fermentation, the soluble COD and total metabolite concentrations under the H_2_&CO_2_ condition led to the highest concentrations of 41.9 ± 4.3 g_COD_ L^−1^_ML_ and 34.5 ± 3.8 g_COD_ L^−1^_ML_, respectively (Figure 2). The CO_2_ condition led to the lowest soluble COD concentration and total metabolite concentrations. Both H_2_ conditions reached similar soluble COD concentrations in the range of 31 g_COD_ L^−1^_ML_ and similar total metabolite concentrations in the range of 22 g_COD_ L^−1^_ML_. The N_2_ reference condition reached a soluble COD concentration of 29.1 ± 1.0 g_COD_ L^−1^_ML_ and a total metabolite concentration of 21.6 ± 0.1 g_COD_ L^−1^_ML_ (Figure 2).

**FIGURE 3:**
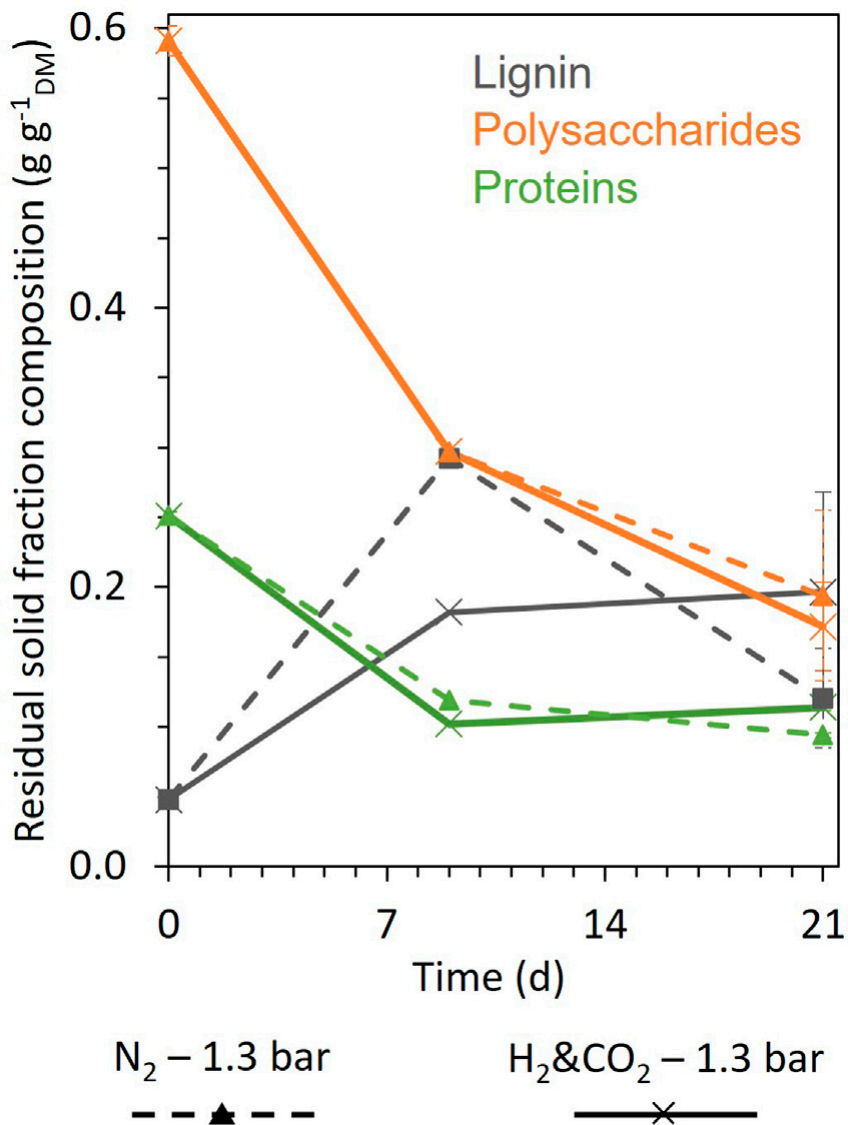
Evolution of the composition of the residual biomass solid fraction on days 0, 9, and 21 for the conditions H_2_ and CO_2_, 1.3 bar (cross, full line), and N_2_, 1.3 bar (triangle, dashed line). Polysaccharides are the sum of all carbohydrates (starch, hemicellulose, cellulose, and soluble carbohydrates). When error bars are not visible, they are present but smaller than the dot symbol; on day 9, no uncertainty bar is shown because the samples were too small to carry out replicates.

Figure 3 compares the evolution of the composition of the residual solid fraction during fermentation for the H_2_&CO_2_ and N_2_ conditions. Starch and soluble carbohydrates (included in the polysaccharide results) were totally consumed over the first 9 days of fermentation, as well as most fermentable proteins and polysaccharides (about 59% and 50%, respectively). After day 9, proteins were consumed much more slowly for both conditions. Further polysaccharide degradation was limited to only about 17% of the initial content. The apparent lignin content increased under both conditions during fermentation. An overestimation of lignin proportion after day 0 is likely due to analytical inaccuracy resulting from limited sample size and the contribution of humic-like substances formed by microbial digestion behaving like lignin in the analysis method. The final solid residue contained 37%–45% of the initial protein content and 29%–33% of the initial polysaccharide content (Figure 3).

### 3.2 Gaseous and soluble metabolite production and consumption

#### 3.2.1 Background fermentation profile of BSG under the N_2_ reference condition

**FIGURE 4:**
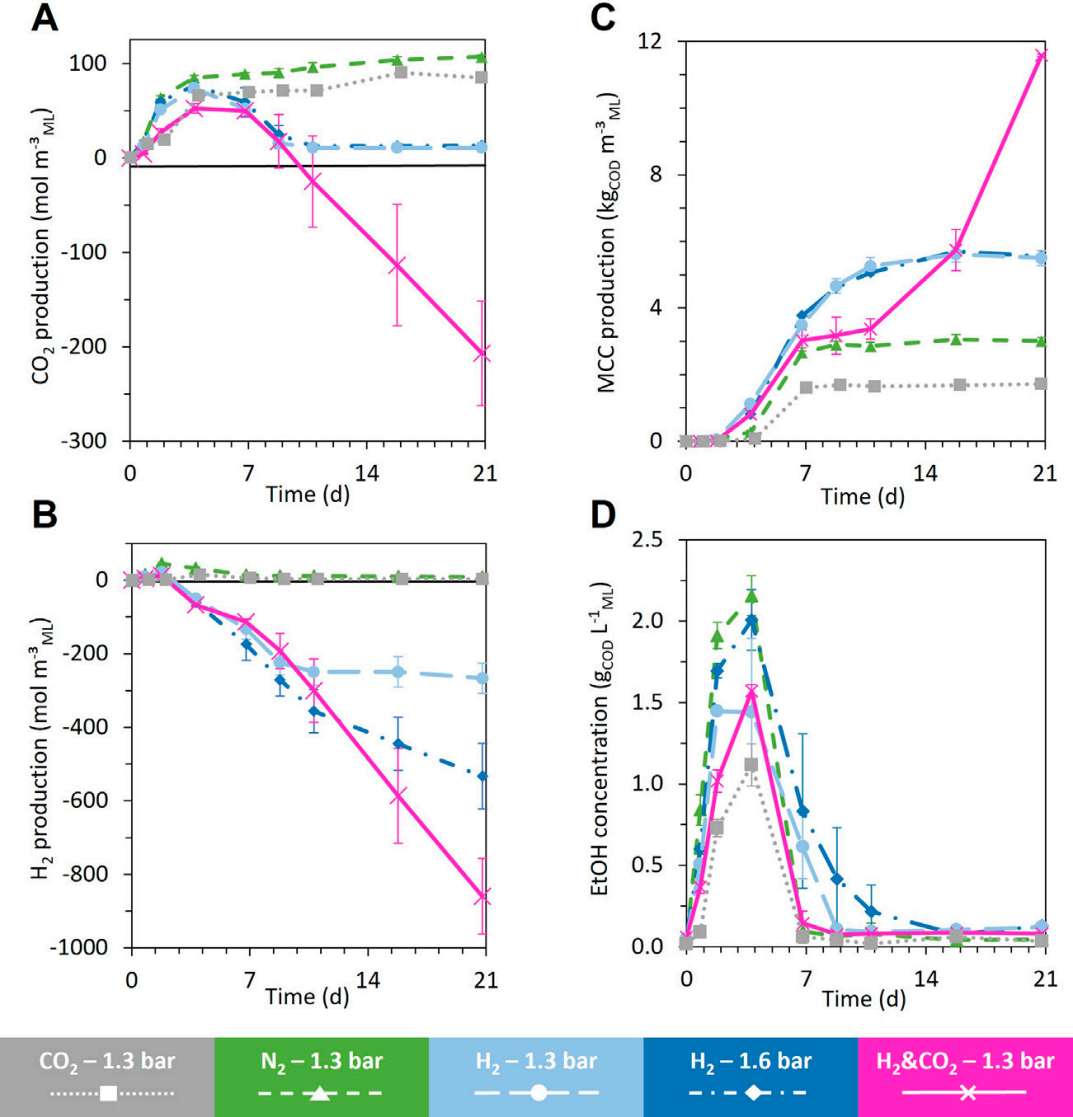
Evolution of CO_2_ production (negative value = consumption) **(A)**, H_2_ production **(B)**, aggregated MCC production [sum of hexanoate (C6), heptanoate (C7), octanoate (C8), nonanoate (C9), decanoate (C10)], **(C)** and ethanol (EtOH) concentration **(D)** during the 21 days fermentation for all the headspace gas conditions, H_2_ and CO_2_, 1.3 bar; H_2_, 1.3 bar; H_2_, 1.6 bar; CO_2_, 1.3 bar; and N_2_, 1.3 bar, respectively, in pink, light blue, blue, gray, and green. When error bars are not visible, they are present but smaller than the dot symbol.

**FIGURE 5:**
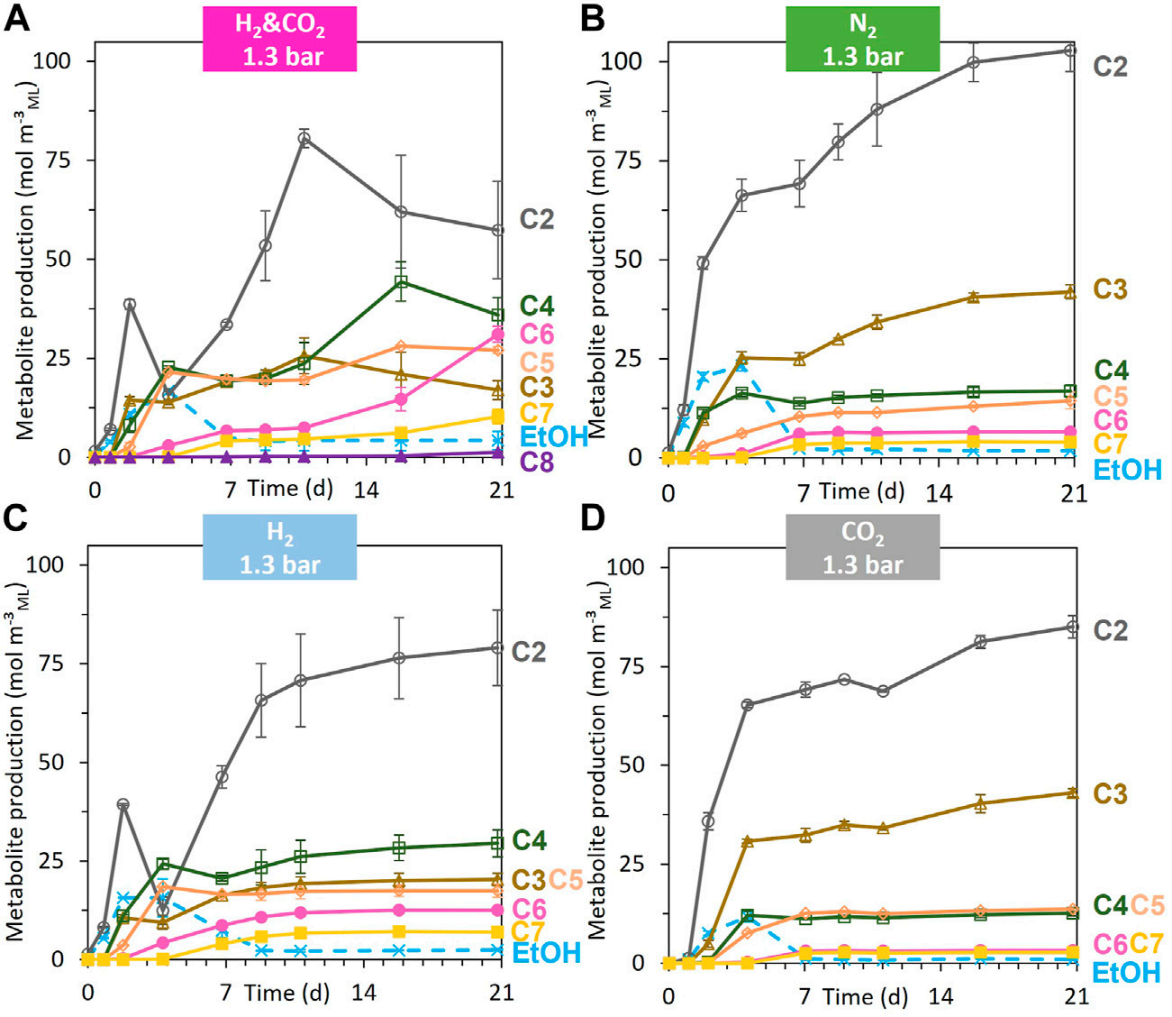
Evolution of the individual metabolite production (negative value = consumption) [acetate (C2), propionate (C3), butyrate (C4), pentanoate (C5), hexanoate (C6), heptanoate (C7), and octanoate (C8)] for the headspace gas condition, H_2_ and CO_2_, 1.3 bar **(A)**; N_2_, 1.3 bar **(B)**; H_2_, 1.3 bar **(C)**; CO_2_, 1.3 bar **(D)** during the 21 days of fermentation. When error bars are not visible, they are present but smaller than the dot symbol.

The acidogenic fermentation of BSG began with a quick and large pH drop during the first two days, accompanied by H_2_ and CO_2_ production (Figures 4A, B; Supplementary Figure S1A). The acidogenic H_2_ was then consumed from day 2 to day 9, unlike acidogenic CO_2_, which did not show a net consumption during the whole fermentation (Figures 4A, B). Alcohols (mainly ethanol reaching a maximal concentration of 2.2 ± 0.2 g_COD_ L^−1^_ML_) and SCC (mainly acetate) were produced from day 0 to day 4 (Figures 4C, D; Supplementary Figure S2). From day 4 to day 7, the ethanol produced during acidogenesis was sharply consumed, simultaneously with the production of MCC (sum of the C6, C7, C8, C9, and C10) (Figure 4C; Figure 5B). MCC were produced only during this period and reached a production of 3.0 ± 0.1 kg_COD_ m^−^³_ML_ (Figure 4C). Hexanoate and heptanoate were the main MCC reaching a production of 1.7 and 1.2 kg_COD_ m^−^³_ML_, respectively (Figure 5B). After day 7 to the end of the fermentation, only a small amount of acetate was produced, accompanied by a slight acidification of the mixed liquor (Figure 5A; Supplementary Figure S1).

**FIGURE 6:**
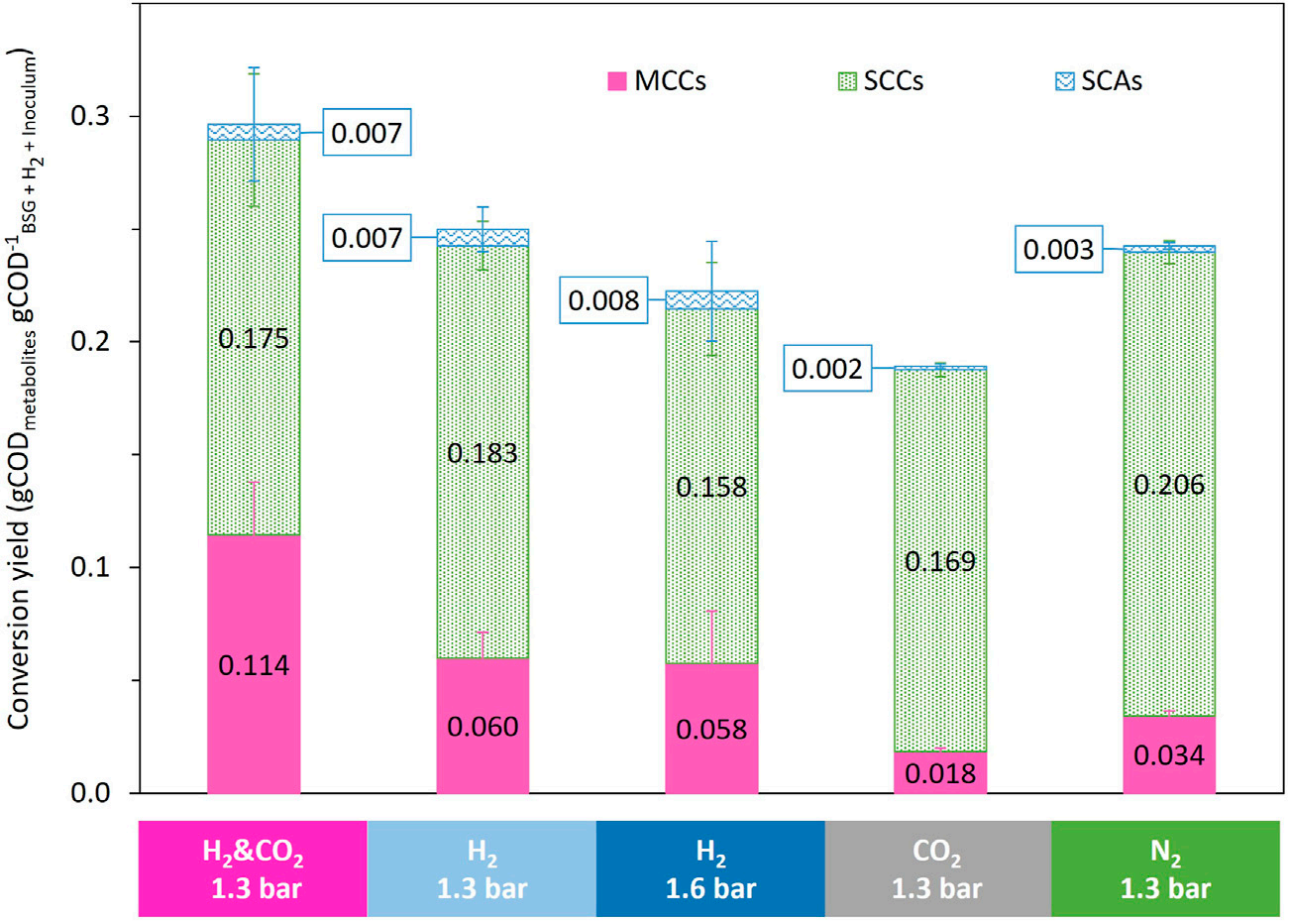
Comparison of the influence of the headspace gas composition on biomass conversion yield to identified metabolites.

At the end of the fermentation, the total metabolites produced by BSG fermentation under N_2_ reached a production of 21.6 kg_COD_ m^−^³_ML_ from the initial concentration of 87 g_COD_BSG_ L^−1^_ML_ and 0.7 g_COD_Inoculum_ L^−1^_ML_, leading to a metabolite/substrate conversion yield of 24.6 ± 0.9% _COD_ (Figure 6; Supplementary Figure S3). The N_2_ reference condition led to an MCC production of 3.0 ± 0.1 kg_COD_ m^−^³_ML_ (Figure 4C). The MCC represented only 14.1 ± 1.1% _COD_metabolites_, whereas SCC represented 84.7 ± 1.4% _COD_metabolites_, mainly as acetate and propionate (Figure 6; Supplementary Figure S4). The final total COD recovered at the end of the fermentation represented more than 88% of the total COD added to the bioreactor (Supplementary Figure S3).

#### 3.2.2 Fermentation profile of BSG supplemented with H_2_, CO_2_, and a mixture of H_2_ and CO_2_

All fermentations started with the same profile as the reference condition under N_2_ (Section 3.2.1), regardless of the nature and pressure of the gas supplied in the headspace. The general trend was a quick and large drop in pH, accompanied by the production of H_2_, CO_2_, ethanol, and SCC within the first four days of fermentation (Figures 4A, B, D, Supplementary Figures S1A, S2). Then, a net consumption of the ethanol and H_2_ occurred concomitantly with a production of MCC until day 7 (Figures 4C, D). After day 7, the fermentation profiles evolved differently depending on the headspace gas nature and pressure.

##### 3.2.2.1 CO_2_ supplementation during BSG fermentation

When only CO_2_ was supplied, the soluble COD concentration, the total metabolites, the production of SCC and MCC, the production of acidogenic H_2_ and CO_2_, and the maximal ethanol concentration during the fermentation were lower than those for the reference condition under the N_2_ atmosphere (Figures 2A, B, Figures 4A–D; Supplementary Figure S2). The total metabolites and the hexanoate, heptanoate, and octanoate production were the lowest of all the tested conditions (Figures 2B, 5D). The MCC production stopped after day 7, when acidogenic H_2_ was no longer consumed, and ethanol concentration decreased below 0.06 g_COD_ L^−1^_ML_, similar to the N_2_ reference condition (Figures 4C, D). After day 7, only a slight increase in C2 and C3 production occurred (Figure 5D).

The addition of CO_2_ in the bioreactor headspace led to a decrease in COD conversion yield to 18.9% _COD_ and an MCC proportion of 9.5% _COD_metabolites_, compared to 24.3% _COD_ and 13.9% _COD_metabolites_ for the N_2_ reference condition (Figure 6). The final total COD recovered at the end of the fermentation represented more than 83.5% of the total COD added to the bioreactor (Supplementary Figure S3).

##### 3.2.2.2 H_2_ supplementation of BSG fermentation

When H_2_ was supplied in the bioreactor headspace, consumption of acidogenic CO_2_ produced during biomass acidogenesis occurred from day 4 to day 11 (Figure 4A). H_2_ consumption stopped completely when no more acidogenic CO_2_ was available in the headspace of bioreactors under 1.3 bar H_2_. The H_2_ consumption rate changed significantly for bioreactors under 1.6 bar H_2_ (Figures 4A, B).

Unlike the reference and CO_2_ conditions, MCC were produced from day 2 to day 11 for both conditions under the H_2_ atmosphere. Net consumption of C2 occurred from day 2 to day 4 without a significant increase in ethanol production but an earlier start in MCC production compared to the reference condition (Figure 5C). From day 7 to day 11, MCC were significantly produced (mainly C6 and C7) (Figure 5C). MCC production stopped on day 11, concomitantly with the exhaustion of acidogenic CO_2_ and when ethanol concentration decreased below 0.2 g_COD_ L^−1^_ML_ (Figures 4A–C). After day 11 and until the end of the fermentation, C2 and C4 were slightly produced for both H_2_ conditions (Figure 5C; Supplementary Figure S5).

The supplementation of H_2_ led to an MCC production of 5.5 ± 0.2 kg_COD_ m^−^³_ML_ and 5.6 ± 0.3 kg_COD_ m^−^³_ML_ for the 1.3 and 1.6 bar conditions, respectively (Figure 4C). Compared to the N_2_ reference condition, the supplementation of H_2_ increased the MCC production by 83% for both conditions. The MCC proportion increased to 23.9% _COD_metabolites_ and 25.8% _COD_metabolites_, respectively, for the 1.3 and 1.6 bar conditions compared to 14.1% _COD_metabolites_ for the reference condition under N_2_ (Figure 6). The SCC proportion decreased to 73.1% _COD_metabolites_ and 70.6% _COD_metabolites_, respectively, for the 1.3 and 1.6 bar conditions compared to 84.7% _COD_metabolites_ for the reference N_2_ condition (Figure 6). The total COD recovered at the end of the fermentation represented more than 96.2% and 90.7% of the total COD added to the bioreactor (COD of added H_2_ included) for the 1.3 and 1.6 bar conditions, respectively (Supplementary Figure S3).

##### 3.2.2.3 H_2_ and CO_2_ mixture supplementation of BSG fermentation

This condition also produced acidogenic H_2_ and CO_2_ at the beginning of the fermentation until days 2 and 7, respectively (Figures 4A, B). All acidogenic H_2_ and CO_2_ were quickly consumed during the following days. H_2_ and CO_2_ were continuously consumed until the end of fermentation.

**FIGURE 7:**
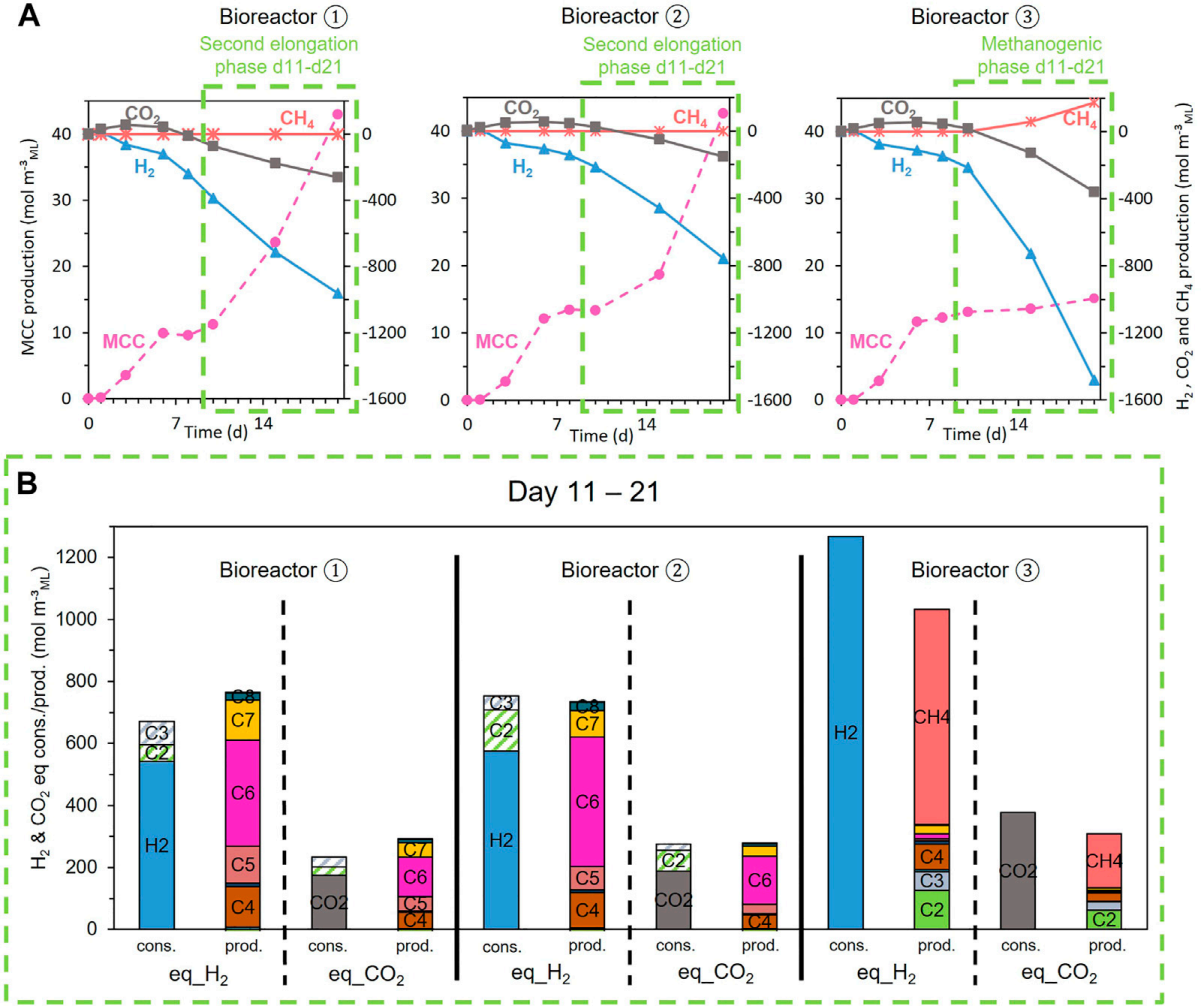
**(A)** Evolution of the CO_2_, H_2_, CH_4_ (right axes, origin on top), and MCC (left axes, origin at the bottom) consumption (negative values) and production (positive values) during the fermentation for each replicate of the H_2_ and CO_2_ condition; **(B)** reducing equivalent and carbon balances expressed in consumed (cons.) and produced (prod.) equivalent_H_2_ and equivalent_CO_2_ during the second phase (green dotted boxes).

The three biological replicates behaved differently. Because methanogenesis was detected in one replicate (bioreactor 3, Figure 7), the latter was excluded from this series and only considered a negative control for the discussion in Section 4.3. A delay in the onset of gas consumption and MCC production (Figure 7A) in the two other bioreactors explains the large standard deviation in Figure 4.

MCC production followed two elongation phases, the first one from day 2 to day 7 and the second one from day 11 to the end of fermentation (Figure 4C). The first phase was concomitant with the ethanol consumption and stopped when the ethanol concentration decreased to 0.2 g_COD_ L^−1^_ML_ (2 mM) on day 7. This kinetic profile is similar to the N_2_ reference condition and the CO_2_ condition (Figure 4D). The MCC production achieved after 7 days of fermentation reached a level intermediate between the MCC production observed under a pure H_2_ atmosphere and pure CO_2_ atmosphere, respectively (Figure 4C). From day 7 to day 11, MCC production stopped, unlike SCC production, which exhibited the highest production of all tested conditions (Figure 4C; Supplementary Figure S2). The second elongation phase started on day 11, whereas the ethanol concentration remained below 0.08 g_COD_ L^−1^_ML_ (0.8 mM) and H_2_ and CO_2_ were consumed (Figures 4A–D). From day 11 to day 16, a simultaneity between the C2 and C3 consumption with the C4, C5, C6, and C7 production could be observed. From day 16 to day 21, C2, C3, C4, and C5 were consumed, whereas C6, C7, and C8 were produced. The final production of C6, C7, and C8 reach 7.9 ± 0.5 kg_COD_ m^-3^_ML_, 3.1 ± 0.5 kg_COD_ m^−3^_ML_, and 0.466 ± 0.006 kg_COD_ m^−3^_ML_, respectively (corresponding to 3.6 ± 0.2 g L^−1^_ML_, 1.3 ± 0.2 g L^−1^_ML_, and 0.191 ± 0.002 g L^−1^_ML_, respectively) (Supplementary Figure S4).

The supplementation of H_2_ and CO_2_ led to an MCC production of 11.6 ± 0.3 kg_COD_ m^−^³_ML_ (Figure 4C). Compared to the reference condition, the supplementation of H_2_ and CO_2_ increased MCC production by 285%. The addition of H_2_ and CO_2_ in the bioreactor headspace enabled a substrate to metabolite COD conversion yields of 29.6% _COD_, compared to 24.3% _COD_ for the reference condition under N_2_ (Figure 6). The MCC proportion increased to 38.5% _COD_metabolites_ compared to 14.1% _COD_metabolites_ for the reference condition under N_2_ (Figure 6). The SCC proportion decreased to 59.1% _COD_metabolites_ compared to 84.7% _COD_metabolites_ for the reference condition under N_2_ (Figure 6). The total COD recovered at the end of the fermentation represented more than 94.7% _COD_ of the total COD added to the bioreactor (COD of added H_2_ included) (Supplementary Figure S3).

### 3.3 H_2_ & CO_2_ condition, second elongation phase: molar consumption ratio

Figure 7A shows that H_2_ and CO_2_ were continuously consumed for the three replicates during the second phase but in different proportions. MCC were continuously produced for two bioreactors (1 and 2) during the second elongation phase, whereas the third bioreactor (3) started methanogenesis without any production of MCC (Figure 7A).

Figure 7B presents the electron and carbon balances in terms of H_2_ (H_2_ = 2 H^+^ + 2 e^−^) and CO_2_ during the second phase (days 11–21). The carboxylates produced or consumed are presented as equivalent H_2_ and CO_2_ (eq_H_2_ and eq_CO_2_), calculated following Eq. 1. Figure 7B shows that electron and carbon balances are nearly closed for each bioreactor.

Bioreactors 1 and 2 have mainly produced MCC (C6, C7, and C8) and have significantly consumed C2 and C3, whereas bioreactor 3 has consumed nearly twice more H_2_ and CO_2_ than the bioreactors 1 and 2 to produce mainly methane without any consumption of SCC (Figure 7B):

$$\left(3n-2\right)\;H_{2}+n\;{CO}_{2}\to {CH}_{3}{\left[{CH}_{2}\right]}_{\left(n-2\right)}COOH+\left(2n-2\right)\;H_{2}O$$ (1)

The H_2_/CO_2_ molar ratio of the exogenous gas mixture supplemented to the bioreactors was 77/23 (%_mol_), which represents supplemented 3.35 mol_H2_ mol^−1^_CO2_. The net consumption of exogenous H_2_ and CO_2_ supplemented in the three bioreactors and the H_2_/CO_2_ molar consumption ratio are summarized in Table 3. Bioreactors 1 and 2 had consumption molar ratios close to 3, whereas bioreactor 3 had a molar ratio very similar to the 3.35 mol_H2_ mol^−1^_CO2_ in the initially supplied gas.

**TABLE 3:** Net H_2_ and CO_2_ consumptions and consumed H_2_/CO_2_ molar ratio during the second phase (days 11–21) for each bioreactor supplemented with H_2_ and CO_2_ in the headspace.

Bioreactor	H_2_ consumption (mol m^−^³_ML_)	CO_2_ consumption (mol m^−^³_ML_)	H_2_/CO_2_ molar consumption ratio (mol_H2_ mol^−1^_CO2_)
1	542	175	3.09
2	576	189	3.05
3	1,269	377	3.36

## 4 Discussion

### 4.1 BSG acidogenic profile until day 11

#### 4.1.1 Most of the BSG fermentable fraction is digested within 11 days

During the first nine days of BSG fermentation, microorganisms have solubilized and partially transformed starch, fermentable proteins, soluble sugar, and 50% of the initially present structural polysaccharides into known metabolites (Figure 3). Under the N_2_ reference condition, on day 11, the total identified metabolite concentration had already reached 86% ± 1% of the final total metabolites achieved after 21 days of fermentation (Figure 2B). After day 11, the remaining part of identified metabolites (only C2 and C3) continued to accumulate but at a much slower rate (Figure 5B). After day 11, the decoupling of the total metabolites and the soluble COD suggests that a non-identified part of the soluble COD is increasing, which could be attributed to further solubilization of BSG components that were not or were hardly fermentable and not converted in the mainstream metabolites covered by our analytical monitoring (Figures 2A, B). The acidogenic CO_2_ produced was not consumed during fermentation, and all the produced acidogenic H_2_ had already been consumed on day 11 (Figures 4A, B). These results suggest that BSG was almost exhausted as an organic substrate within the first 11 days of fermentation and that most BSG fermentable fractions were transformed into known metabolites.

The residual solid matter and the soluble COD concentration evolved in a similar way for both the H_2_ & CO_2_ and the reference N_2_ conditions (Figures 2A, 3). These results suggest that BSG solubilization by the microorganisms was independent of the gas supplied. This does not agree with Arslan et al. (2012), who observed a decrease in the hydrolysis degree of potato wastes after 1 week of fermentation when a mixture of H_2_ and CO_2_ (1:1%_mol_) replaced the N_2_ in the headspace.

Nevertheless, a significant increase of 60% higher total metabolites was observed on day 21 when both H_2_ and CO_2_ were supplied compared to the N_2_ reference condition (Figure 2B). This increase in identified metabolites occurred essentially after day 9 and can be attributed to H_2_ and CO_2_ conversion to soluble metabolites (see Section 4.2).

Starting from BSG as a unique feedstock, the metabolite production obtained after 9 days of fermentation is similar to those obtained by Liang and Wan (2015), who obtained approximately 90% of the final total metabolites in 10 days at pH 7. Perimenis et al. (2018) obtained 90% of the maximal volatile fatty acid concentration in 15 days.

#### 4.1.2 BSG feeds a first chain elongation phase (days 2–11) through acidogenic ethanol and H_2_ consumption

Under all tested conditions, MCC production began slowly from day 2 to day 4, with the main production occurring between day 4 and day 7 (Figure 4C). During the first elongation phase, the MCC production was concomitant with ethanol and H_2_ consumption. It stopped when ethanol concentration decreased below 0.2 g_COD_ L^−1^_ML_ (2 mM) and when the produced acidogenic H_2_ was consumed (Figures 4B–D).

Under the N_2_ reference condition, the distinctly consumed COD_ethanol_ was equivalent to 68% of the total COD_MCC_ produced. The acidogenic H_2_ consumed represented 19% of the total COD_MCC_ produced. Ethanol and H_2_ correspond to 87% of the total produced COD_MCC_. Some of the acidogenic ethanol and produced H_2_ may have been directly converted to MCC and thus not monitored, which could further increase these COD ratios. Most probably, the remaining non-identified 13% of the total COD_MCC_ could be related to the elongated SCC. This is consistent with the kinetic and thermodynamic study of González-Cabaleiro et al. (2013), which evidenced the feasibility of the production of C6 (or MCC) through the reduction of acetate or butyrate with ethanol but also based on ethanol only. Some parts of the consumed H_2_ COD could have contributed to the production of SCC by acetogens (Bengelsdorf et al., 2018).

Lactate could also have been produced in the early days of the BSG acidogenesis stage and used as acetyl-CoA and reduced cofactor precursors to generate MCC through lactate-based RBO (Zhu et al., 2015; Liu et al., 2020). The lactate-based RBO generates 1 mole of CO_2_ for each mole of pyruvate decarboxylated to acetyl-CoA (Cavalcante et al., 2017). For the N_2_ reference condition, Figures 4A, C show that CO_2_ production stopped after day 4 concomitantly with a slight production of MCC to reach 0.3 kg_COD_ m^−^³_ML_. This slight increase in MCC production could be due to lactate-based RBO. The production of MCC from lactate could have occurred during the first 4 days of fermentation but does not appear to be a dominant MCC production route. These assumptions are consistent with the observed CO_2_ evolution. However, they cannot be definitely demonstrated as CO_2_ can be involved in several simultaneous biological and physicochemical processes. Moreover, this slight increase could also result from the start of ethanol-based RBO as ethanol concentration reached its maximum on day 4 and started to be distinctly consumed afterward.

These results suggest that ethanol produced by the BSG acidogenesis is the main electron donor and carbon source for the first chain elongation phase. Acidogenic H_2_ might also play a role in the production of MCC. These results also suggest that both ethanol concentration and H_2_ partial pressure could be limiting factors for MCC production.

#### 4.1.3 Supplying CO_2_ and H_2_ affects the CE process

##### 4.1.3.1 Supplying CO_2_ alone inhibits the whole fermentation and ethanol-based CE pathway

When only CO_2_ was exogenously supplied to the culture broth, the total metabolite, including the produced MCC, was the lowest of all tested conditions (Figures 2B, 4). The stronger acidification of the medium observed for the CO_2_ condition (Supplementary Figure S1) compared to the other conditions possibly resulted from an insufficient buffer capacity of the BSG and mineral medium to resist both the acidogenic fermentation and the solubilization of CO_2_. Although the first acidification step lasted less than 16 h, a selection of more acid-resistant microorganisms at the beginning of fermentation could have occurred. This selection could explain the lower total metabolite production. Moreover, the lack of a reductive atmosphere could explain the poor MCC production.

Compared to the other non-reductive condition under the N_2_ atmosphere, the maximal ethanol concentration achieved for the CO_2_ condition was two times lower (Figure 4D). Excessive ethanol oxidation could have been promoted in these acidic and non-reductive conditions with low H_2_ partial pressure (Roghair et al., 2018). Ethanol-oxidizing microorganisms could have oxidized ethanol to acetate, decreasing the ethanol availability for the ethanol-based RBO pathway. This could explain the low MCC production under this condition.

Different effects of CO_2_ supplementation during complex organic feedstock fermentation were reported in the literature. The present study shows that CO_2_ supplementation inhibits the entire BSG fermentation and does not select any specific metabolite production. However, Arslan et al. (2012) and Arslan et al. (2013) observed that CO_2_ drives the organic waste stream fermentation to the production of n-butyrate. Conversely, Darvekar et al. (2019) reported that from lignocellulosic substrates, CO_2_ supplementation decreased the total metabolite production and moved carboxylate production toward MCC production.

##### 4.1.3.2 Supplying H_2_ alone promotes CE and allows acidogenic CO_2_ consumption

Compared to the N_2_ reference condition, the supply of H_2_ during the BSG fermentation allowed a quicker start in MCC production and nearly doubled the final amount produced (Figure 4C). Thus, H_2_ played an important role in MCC production from raw biomass. When supplying H_2_, the H_2_ consumption was concomitant to acidogenic CO_2_ consumption and MCC production. Elongation stopped when acidogenic CO_2_ was exhausted and ethanol concentration reached 0.06 g_COD_ L^−1^_ML_ (or 0.625 mM) (Figures 4A–D).

On the one hand, H_2_ can feed the elongation process through acetate reduction to ethanol (Steinbusch et al., 2008; González-Cabaleiro et al., 2013; Spirito et al., 2014). Compared to the N_2_ control condition, a net consumption of acetate occurred when H_2_ was supplied, but no increase in ethanol concentration during this period was detected (Figures 4D, Figures 5B, C). As the MCC production started before the N_2_ reference condition, this potential H_2_- and acetate-based ethanol could be directly converted to MCC, resulting in no detectable increase in ethanol production. This could partially explain the increase in MCC production but does not explain the consumption of acidogenic CO_2_.

On the other hand, the MCC production was concomitant with the acidogenic CO_2_ and H_2_ consumption (Figures 4A–C). This suggests that acidogenic CO_2_ could be linked to the MCC production probably through homoacetogenesis and the resulting acetate and/or acetyl-CoA production (Section 4.3).

The results obtained for both H_2_ conditions (with a H_2_ partial pressure of 1.3 and 1.6 bar) are similar in terms of the acidogenic and elongation phases. Compared to the results of Steinbusch et al. (2008) obtained at pH 5.0, in this study at pH 7.0, the sole increase in H_2_ partial pressure from 1.3 to 1.6 bar did not increase the reduction of carboxylates to their corresponding alcohols nor allowed a better MCC production (Figures 4C, 6; Supplementary Figure S4). In other words, at pH 7.0, the single increase in H_2_ partial pressure did not stimulate the microbial reduction of acetate to ethanol or its direct assimilation to produce elongated carboxylates. Nevertheless, for both H_2_ conditions, H_2_ consumption stopped concomitantly with the MCC production when no more CO_2_ was available. Therefore, the CO_2_ availability in the headspace could be the limiting factor for MCC production when H_2_ is supplied.

### 4.2 H_2_ and CO_2_ together feed a second elongation phase after day 11

After day 11, both H_2_ and CO_2_ continued to be consumed, as well as acetate, but the ethanol concentration remained at a concentration below 0.08 g_COD_ L^−1^_ML_ (0.8 mM), while the production of MCC restarted (Figures 4A–D, Figure 5A). Considering that the BSG solubilization and fermentation were mainly exhausted within the first 9–11 days of fermentation (see above) and that ethanol concentration remained around 1 mM, the recovery of MCC production after day 11 would be fed only by the exogenous H_2_ and CO_2_ supply.

During this second elongation phase (days 11–21), if H_2_ and CO_2_ are considered the only reactants for carboxylate production, then Eq. 1 can be considered. In addition, it can be assumed that all the moles of carboxylates produced or consumed during this period can be expressed as their H_2_ molar equivalent and CO_2_ molar equivalent, following the stoichiometry of Eq. 1. Following these assumptions, electron and carbon balances were calculated in terms of eq_H_2_ and eq_CO_2_ from the produced and consumed carboxylates. Figure 7B shows that almost all the net consumed H_2_, CO_2_, and carboxylates are recovered in terms of eq_H_2_ and eq_CO_2_ in the produced carboxylates for replicates 1 and 2. Balances are not perfectly closed, but it can be assumed that other microbial activities misroute the electron equivalents from H_2_ and carbon from CO_2_ toward microbial cell synthesis and other unknown organic end-products. Moreover, even if most of the BSG fermentable fraction was solubilized, some residual soluble fraction could also be transformed into metabolites. Nevertheless, as the balances are quite complete, the later processes can only be considered secondary.

The increase in MCC production by 285% when both H_2_ and CO_2_ are supplied to the microorganisms compared to the N_2_ reference condition was consistent with the observations made by Darvekar et al. (2019), who showed that the final concentration of C6 was 186% higher when both H_2_ and CO_2_ were supplied [H_2_:CO_2_ (1:1)] than the N_2_ reference condition.

### 4.3 Consumed H_2_/CO_2_ molar ratio of 3 as a signature of CE based on H_2_, CO_2_, and SCC

Table 3 presents H_2_/CO_2_ molar ratios of consumed H_2_ and CO_2_ for each replicate from day 11 to the end of fermentation. Reactors 1 and 2 hosted a second elongation phase (Figure 7A), with H_2_/CO_2_ molar ratios of 3.09 and 3.05, respectively (Table 3). Table 4 presents the H_2_/CO_2_ molar ratio expected following Eq. 1. According to Eq. 1, the H_2_/CO_2_ consumption molar ratio depends on the number of carbon atoms (*n*) of the produced carboxylates and never reaches the value of 3. In other words, as electron and carbon balances are nearly closed and the H_2_/CO_2_ molar ratio exceeds 3, the carboxylate production cannot be explained by the stoichiometry of Eq. 1.

**TABLE 4:** The H_2_/CO_2_ molar ratio following Eq. 1 depends on the number of carbon atoms in the produced carboxylate.

Carboxylate	C2 (*n* = 0)	…	C6 (*n* = 4)	…	C10 (*n* = 8)
Molar ratio H_2_/CO_2_ (mol_H2_ mol^-1^_CO2_)	4/2 = 2	…	16/6 = 2.66	…	28/10 = 2.8

Figure 5A shows a net consumption of SCC (i.e., C2, C3, C4, and C5) concomitantly with the production of MCC (C6, C7, and C8), without detectable net consumption of ethanol during the second elongation phase. These SCC are then expected to be part of the reactants in addition to H_2_ and CO_2_. If there is a simultaneous consumption of SCC, H_2_, and CO_2_ with an H_2_/CO_2_ molar ratio of 3 during the production of MCC, then Eq. 2 can be considered.

Eq. 2 does not exclude that H_2_ and CO_2_ are first converted to ethanol following Eq. 3 as ethanol production from H_2_ and CO_2_ is thermodynamically favorable (ΔG° = −97.6 kJ mol^−1^_ethanol_) and follows an H_2_/CO_2_ consumption molar ratio of 3 (Eq. 3). Therefore, the second elongation phase can be ethanol-based, assuming a low pseudo-steady-state concentration of extracellular ethanol, as it could be consumed as fast as it was produced. Ethanol can contribute to elongating SCC (Eq. 4) or be directly converted to carboxylates (Eq. 5):

$$3n\;H_{2}+n\;{CO}_{2}+\left(n/2\right)\;{CH}_{3}{\left[{CH}_{2}\right]}_{m}COOH\to \left(n/2\right){CH}_{3}{\left[{CH}_{2}\right]}_{\left(m+2\right)}COOH+2n\;H_{2}O$$ (2)

$$3n\;H_{2}+n\;{CO}_{2}\to \left(n/2\right)\;{CH}_{3}{CH}_{2}OH+\left(3n/2\right)\;H_{2}O$$ (3)

$$\left(n/2\right){CH}_{3}{CH}_{2}OH+\left(n/2\right)\;{CH}_{3}{\left[{CH}_{2}\right]}_{\left(m+2\right)}COOH\to \left(n/2\right)\;{CH}_{3}{\left[{CH}_{2}\right]}_{\left(m+2\right)}COOH+\left(n/2\right)H_{2}O$$ (4)

$$\left(n/2\right){CH}_{3}{CH}_{2}OH\to {CH}_{3}{\left[{CH}_{2}\right]}_{\left(n-2\right)}COOH+\left(\left(n/2\right)-2\right)H_{2}O+2\;H_{2}$$ (5)

All these reactions are consistent with the Wood–Ljungdahl pathway, which consumes H_2_ and CO_2_ to produce acetyl-CoA. Acetyl-CoA can be the precursor of ethanol or acetate and can be directly used in the RBO pathway for acyl-chain elongation (Bengelsdorf et al., 2018). The H_2_ reductive atmosphere could maintain a sufficiently high NADH/NAD^+^ ratio for activating the RBO pathway, which will regenerate the NAD^+^ oxidized cofactor (Rodríguez et al., 2006; Spirito et al., 2014). A positive ATP balance could be reached considering that ATP generation is due to the electron-transport phosphorylation occurring with the electro-chemical gradients (H^+^ and Na^+^) across the cytoplasmic membrane linked to the reduction of NAD^+^ (Rodríguez et al., 2006; González-Cabaleiro et al., 2013; Liew et al., 2016).

**FIGURE 8:**
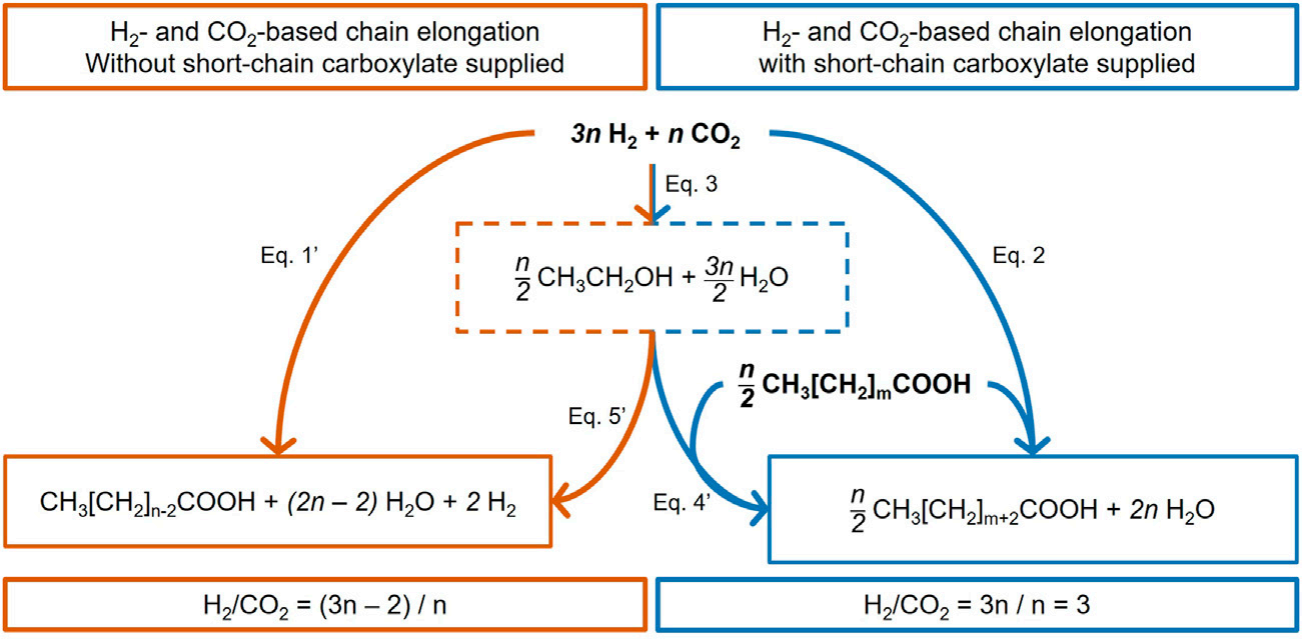
Stoichiometric balances of the H_2_ and CO_2_ conversion that can lead to MCC, either as the only reactant (left, orange pathway) or supplemented with shorter-chain carboxylates (right, blue pathway). *n* is a multiple of 2. Eq. 1', Eq. 4', and Eq. 5' are stoichiometrically equivalent to Eq. 1, Eq. 4, and Eq. 5, respectively (Supplementary Excel).

**FIGURE 9:**
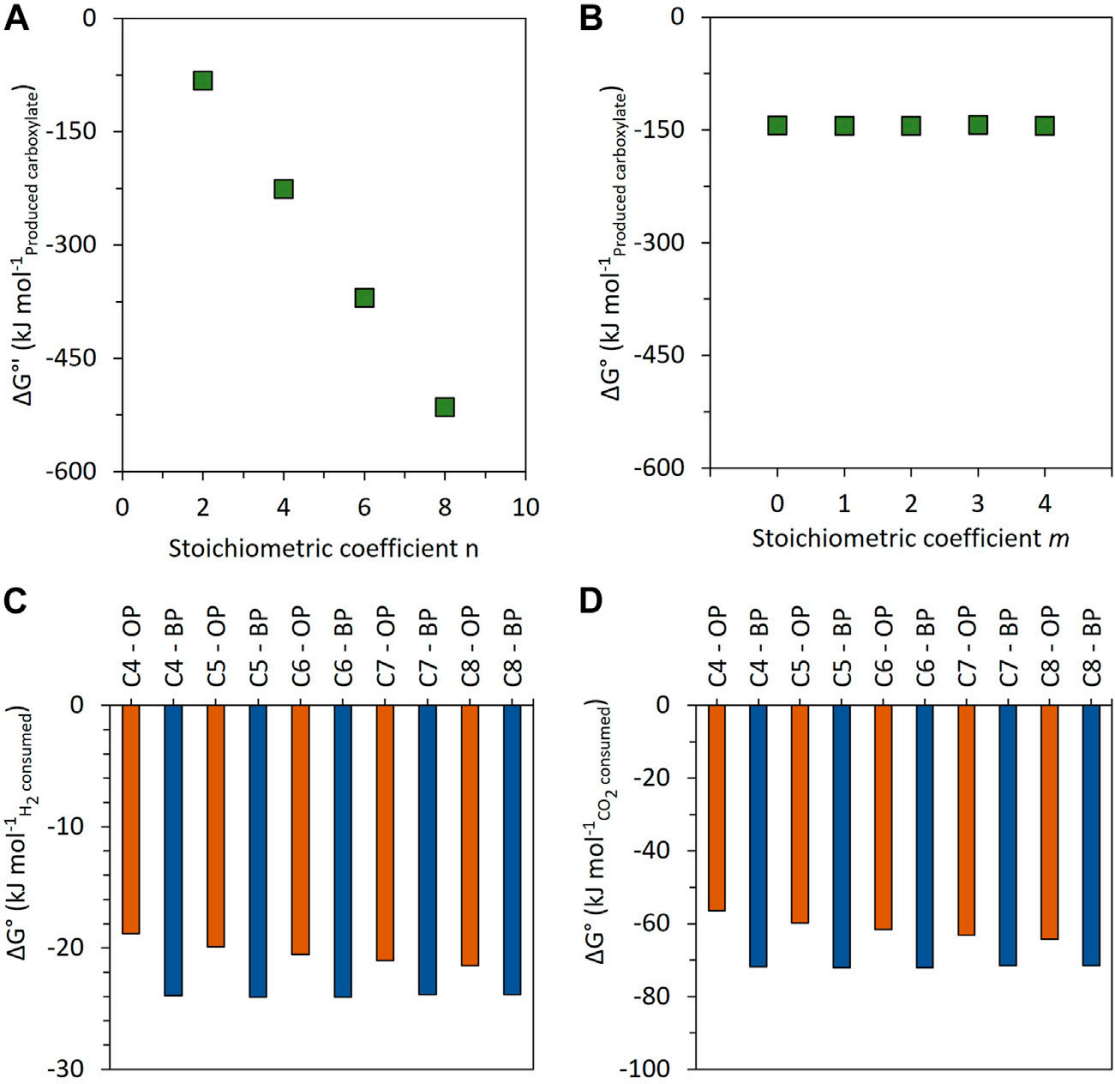
Change in standard Gibbs free energy for carboxylate production as a function of *n* (without shorter-chain carboxylate consumption—orange pathway of Figure 8) **(A)** and as a function of *m* (with shorter-chain carboxylate consumption (*n* = 2)—blue pathway of Figure 8) **(B)**. Comparison of the change in Gibbs free energy for carboxylate production (C4, C5, C6, C7, and C8) between the orange pathway (OP) and the blue pathway (BP), normalized by the consumed H_2_ **(C)** and consumed CO_2_ **(D)**.

Figure 8 summarizes the thermodynamically feasible elongation reactions based on H_2_ and CO_2_. On the right side, the blue reactions describe the *n*-carbon elongation of initially present carboxylates. This pathway can directly use *3n* H_2_, *n* CO_2_, and *n/2* carboxylate for carboxylate elongation (Eq. 2) or ethanol as a metabolic intermediate (Eq. 4'). On the left side of Figure 8, the orange reactions are based only on H_2_ and CO_2_ as reactants with no consumption of shorter carboxylates for carboxylate elongation, passing by ethanol as a metabolic intermediate (Eq. 5') or not (Eq. 1'). Eq. 1', Eq. 4', and Eq. 5' are stoichiometrically equivalent to Eq. 1, Eq. 4, and Eq. 5, respectively (Supplementary Excel). Both the orange pathway (Figure 9A; function of *n*) and the blue pathway (Figure 9B; function of *m*, with *n = 2*) are thermodynamically favorable.

The orange reactions of Figure 8 (Eq. 1' or the sequence of Eq. 3 + Eq. 5') show a ΔG° increasingly favorable with increasing the *n* stoichiometric value (Figure 9A). However, they do not explain the experimentally measured H_2_/CO_2_ consumption molar ratios of 3.05 and 3.09 (Table 3). Indeed, the stoichiometric ratio of the orange reactions is *n*-dependent due to the presence of H_2_ on the product side. This is consistent with the first paragraph of Section 4.3. Therefore, the observed carboxylate production cannot be explained by the stoichiometry of the orange reactions.

The blue reactions of Figure 8 describe the elongation of initially present carboxylate to elongated carboxylate from H_2_ and CO_2_ as reactants, with (Eq. 4') or without (Eq. 2) ethanol as the identified intermediate. These reactions are consistent with the RBO pathway for a stoichiometric coefficient *n* equal to a multiple of 2. Stoichiometrically, the elongation of *n* moles of carboxylates needs *3n* moles of H_2_ and *n* moles of CO_2_ and gives a constant value of 3 as the stoichiometric H_2_/CO_2_ ratio consumed (*3n*/*n* = 3). This stoichiometry is consistent with the experimentally measured H_2_/CO_2_ molar ratio in this study (Table 3). These reactions are thermodynamically feasible and show a constant value of ΔG° = −143.9 ± 0.6 kJ mol^−1^_carboxylate_, independent of *m*, the carbon number in the initial carboxylate elongated (Figure 9B).

Thermodynamically, when comparing the carboxylate elongation of two carbon atoms from H_2_ and CO_2_, either with already formed carboxylates (blue pathway) or without carboxylates as a precursor (orange pathway), the changes of standard Gibbs free energy per mole of consumed H_2_ (Figure 9C) and per mole of consumed CO_2_ (Figure 9D) show that the blue pathway is more thermodynamically favorable. Therefore, it can be expected to be preferred by microorganisms. Altogether, the stoichiometric and thermodynamic analyses of the second elongation phase are consistent with a chain elongation based on the initially present carboxylates and with H_2_ and CO_2_ consumption. Nevertheless, it cannot be clarified whether this pathway passes or not through ethanol as a metabolic intermediate [(Eq.3 + Eq.4') vs. (Eq.2)].

This elongation based on SCC, H_2_, and CO_2_ can also explain the increase in MCC production observed in this study when only H_2_ was supplied to the microorganisms (Section 4.1.3.2). Indeed, the supplementation of H_2_ allowed acidogenic CO_2_ to be consumed and can explain a nearly doubled production of MCC compared to the N_2_ reference condition. As the elongation stopped concomitantly with the lack of CO_2_, this confirms the crucial role of both H_2_ and CO_2_ in mixed microbial chain elongation with or without supplementation of organic feedstock.

More details on the potential SCC-, H_2_-, and CO_2_-driven elongation pathway catalyzed by microbial communities would be obtained by carrying long-term cyclic, fixed-volume semi-batch fed reactor fermentation with H_2_ and CO_2_ supply, with or without organic feedstock addition. Other challenges to overcome are the full inhibition of methanogenesis and increasing the concentration of the produced MCC while maintaining a stable production without inhibition of the microorganisms due to the MCC toxicity. The analysis of microbial community dynamics during the fermentation will provide new insights into the MCC production process and if different microbial populations are responsible for the different detected fermentation profiles.

## 5 Conclusion

Two elongation phases were distinguished when organic feedstock, H_2_, and CO_2_ were fed together to microorganisms. The first phase seems mainly based on the ethanol conversion generated by the acidogenic phase. The second elongation phase occurred when the organic feedstock was almost completely consumed. Further metabolite production could only result from the consumption of the H_2_ and the CO_2_ supplied to the system.

Based on the carbon and reducing equivalent balances, thermodynamic calculations, and a specific H_2_/CO_2_-molar consumption ratio of 3 mol_H2_/mol_CO2_ (experimentally measured and stoichiometrically confirmed), this study suggests that the second elongation phase results from the elongation of accumulated SCC fed by H_2_ and CO_2_. According to our knowledge, this is the first time that an elongation phase based only on SCC, H_2_, and CO_2_ with such a well-determined H_2_/CO_2_ molar consumption ratio is described. Ethanol can be a non-detected intermediate metabolite synthesized from H_2_ and CO_2_, contributing to the elongation of accumulated SCC. This process also fits an H_2_/CO_2_ molar ratio of 3. However, ethanol could not act as the only intermediate reactant as the stoichiometry of ethanol-only elongation does not fit the observed H_2_/CO_2_ molar ratio of 3.

When various reducing equivalent and carbon sources are supplied to microorganisms, MCC production profiles are more difficult to interpret because of diverse metabolic pathways occurring simultaneously. Nevertheless, the supplementation of H_2_ during the organic substrate conversion allowed acidogenic CO_2_ to be consumed and nearly doubled the MCC production. This confirms that MCC production from organic feedstock fermentation is also dependent on H_2_ and CO_2_ consumption.

## Data Availability

The raw data supporting the conclusion of this article will be made available by the authors, without undue reservation.

## Abbreviations

BSG, brewer’s spent grain; CE, chain elongation; COD, chemical oxygen demand; CRAW, Walloon agricultural research center; EtOH, ethanol; FM, fresh matter; MCC, medium-chain carboxylates; ML, mixed liquor; RBO, reverse β-oxidation; SCA, short-chain alcohols; SCC, short-chain carboxylates; TS, total solid; VS, volatile solid; PropOH, propanol; ButOH, butanol; C2, acetate/acetic acid; C3, propionate/propionic acid; C4, butyrate/butyric acid; iC4, isobutyrate/isobutyric acid; C5, pentanoate/pentanoic acid; iC5, isopentanoate/isopentanoic acid; C6, hexanoate/hexanoic acid; C7, heptanoate/heptanoic acid; C8, octanoate/octanoic acid; C9, nonanoate/nonanoic acid; C10, decanoate/decanoic acid; PhC2, phenylacetate/phenylacetic acid; PhC3, phenylpropionate/phenylpropanoic acid.
