# Supplementary material for: A specific H2/CO2 consumption molar ratio of 3 as a signature for the chain elongation of carboxylates from brewer’s spent grain acidogenesis
Source: Front Bioeng Biotechnol. 2023 Jun 1;11:1165197. doi: 10.3389/fbioe.2023.1165197 (PMC10267453; doi:10.3389/fbioe.2023.1165197)
Supplement: Supplementary file 2 [file DataSheet1.docx]

Supplementary Material

# Supplementary Table

**Table S1.** Mass - COD - mole equivalences for the metabolites considered in the investigated conversions. Molecular weight (MW), stoichiometric chemical oxygen demand (ThCOD calculated following **Eq. S1**) and stoichiometric equivalences as H_2_ (eq_H_2_) and CO_2_ (eq_CO_2_)) following **Eq. 1** for carboxylates and **Eq. S2** for alcohols, and the resulting conversion factors used (g_COD_ mol^-1^ and g_COD_ g^-1^). Detailed calculation can be found in the conversion table sheet of the **Supplementary Excel file**.

**Table S2.** Unit conversion table.

**Table S3.** Standard Gibbs free energy change of formation table used for calculations.

# Supplementary Figures


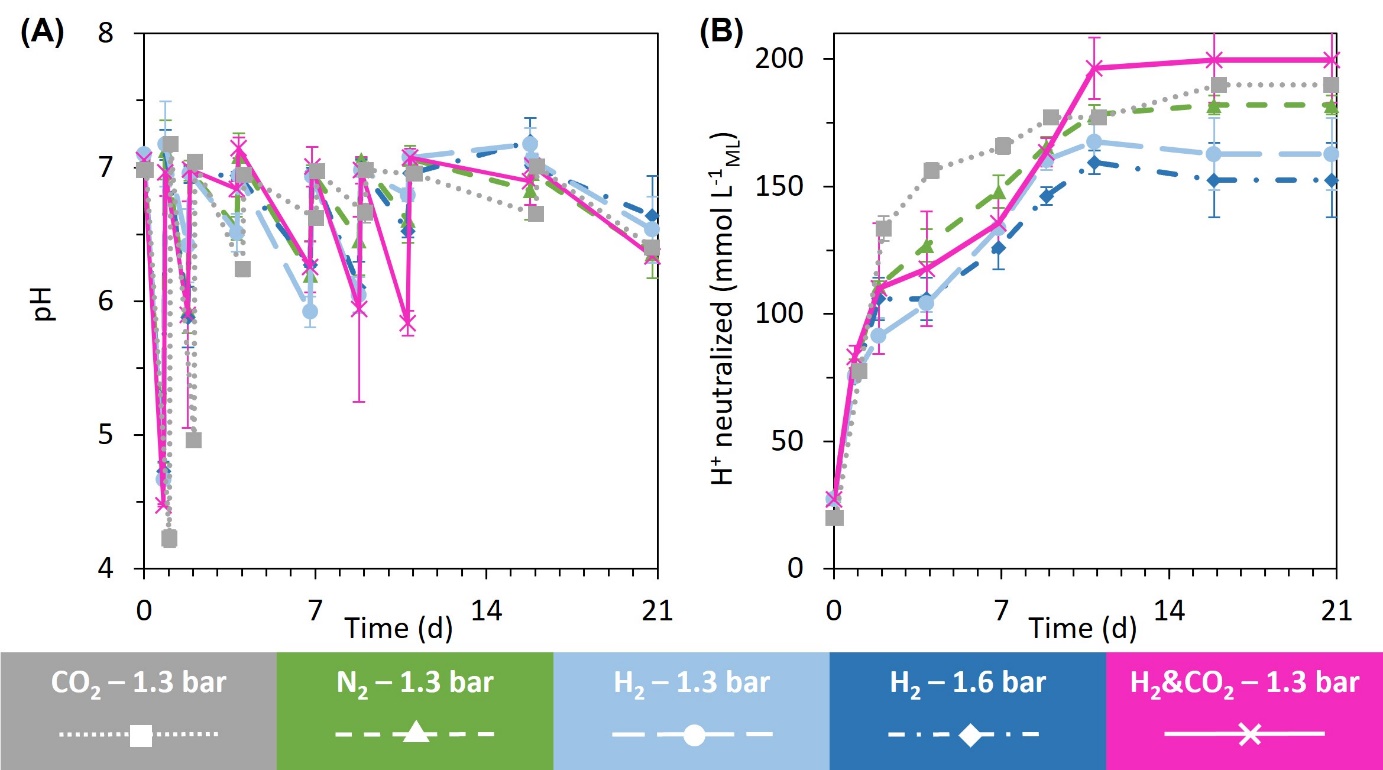


**Fig. S1.** Evolution of pH (A) and neutralized H^+^ (B) during the 21 days of fermentation under the different conditions tested.


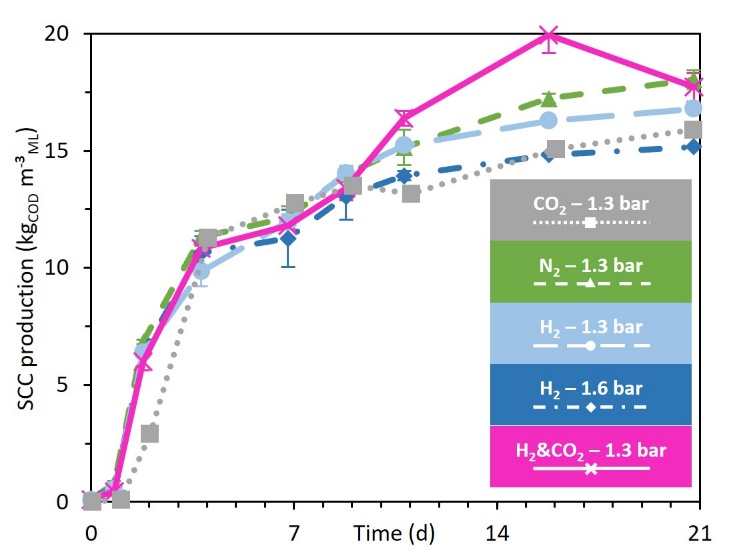


**Fig. S2.** Evolution of the short-chain carboxylates (SCC) production during the 21 days of fermentation under the different conditions tested.


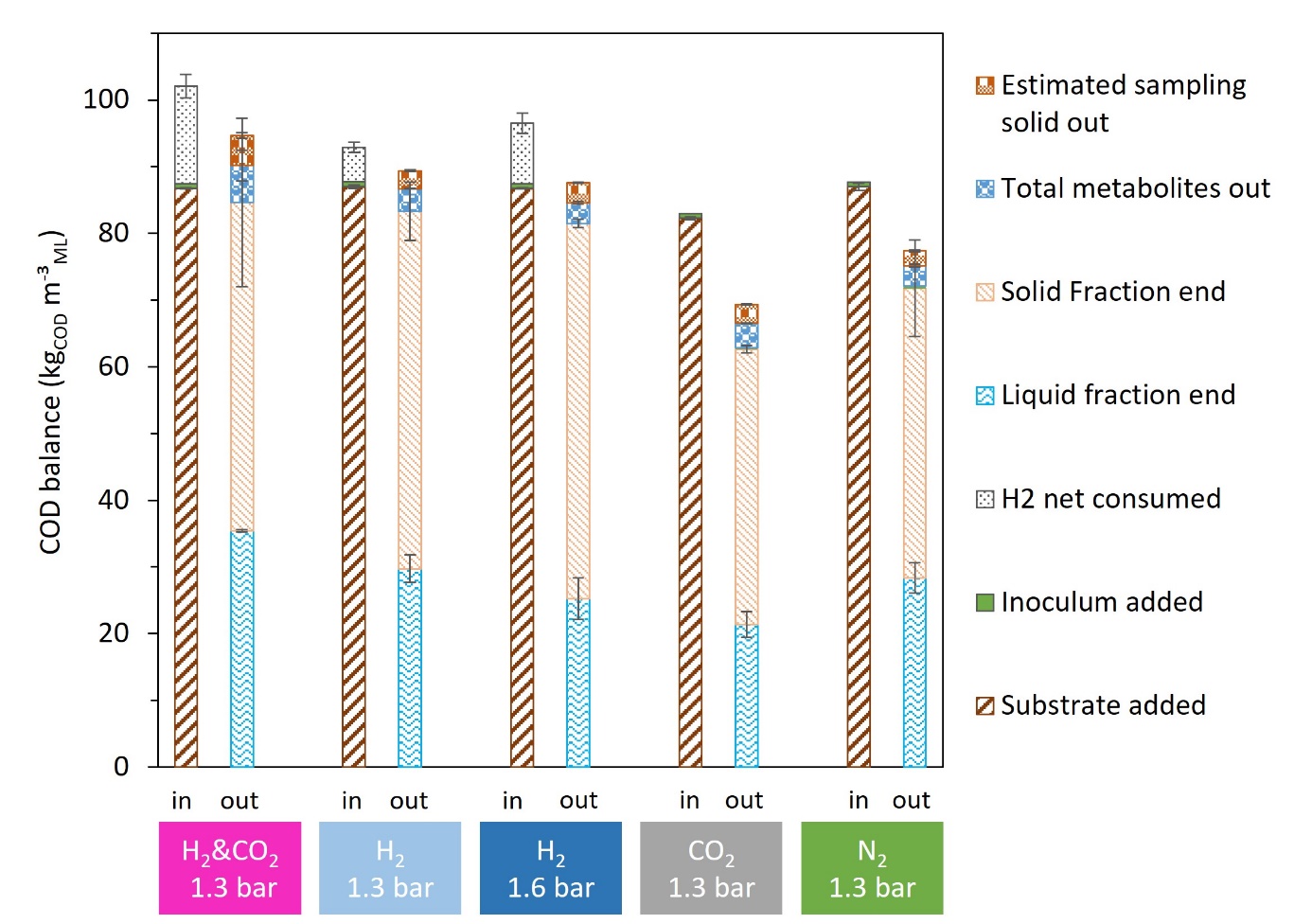


***Fig. S3.*** *COD balance between the input (in: initial BSG substrate, inoculum, H_2_ supplied and consumed during fermentation) and the output (out: extracted samples during the fermentation (Estimated sampling solid out (****Eq. S2****)), total metabolites out and recovered at the end of the fermentation (liquid fraction end and solid fraction end) of the bioreactors.*


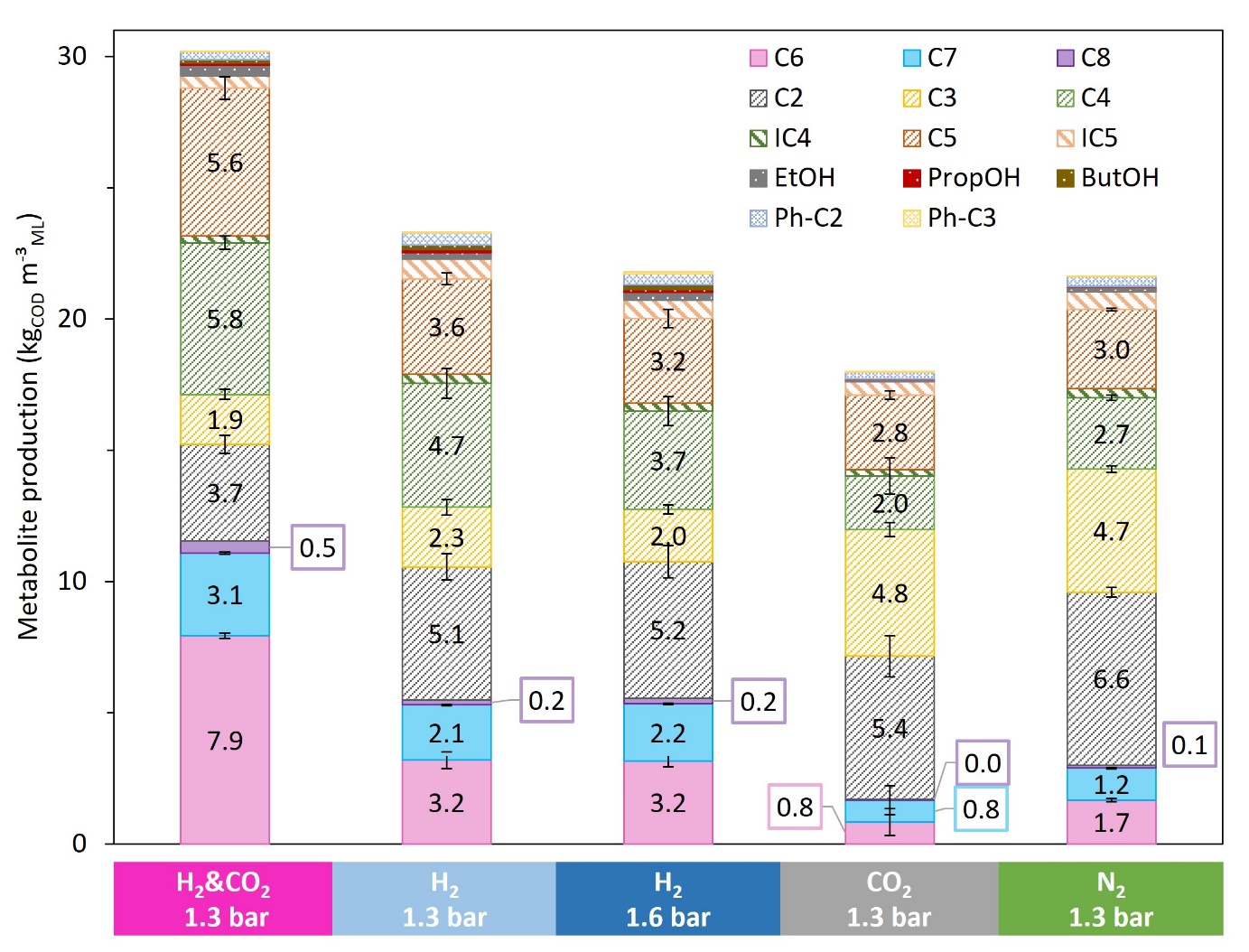


**Fig. S4.** Metabolite production profile after 21 days of fermentation under the different conditions tested.


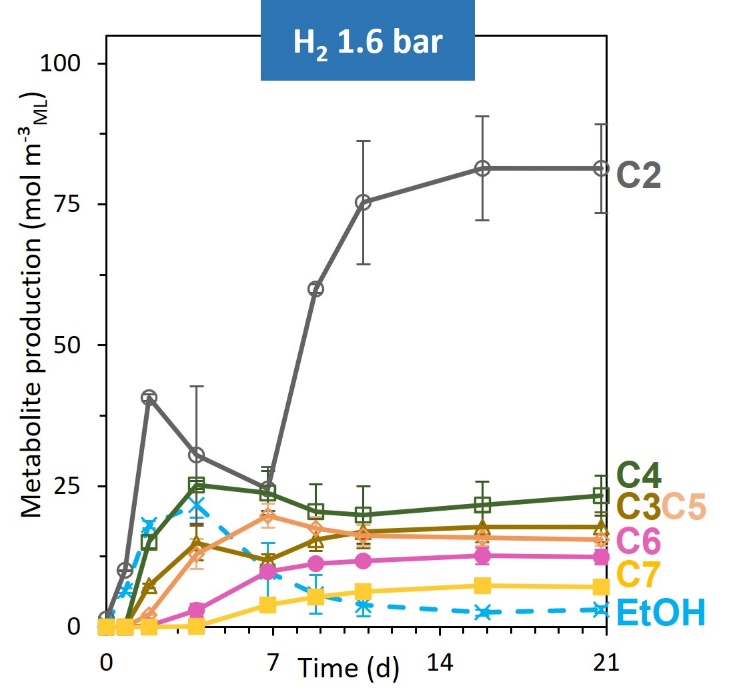


**Fig. S5.** Evolution of individual metabolite productions for the condition under H_2_ adjusted at 1.6 bar.


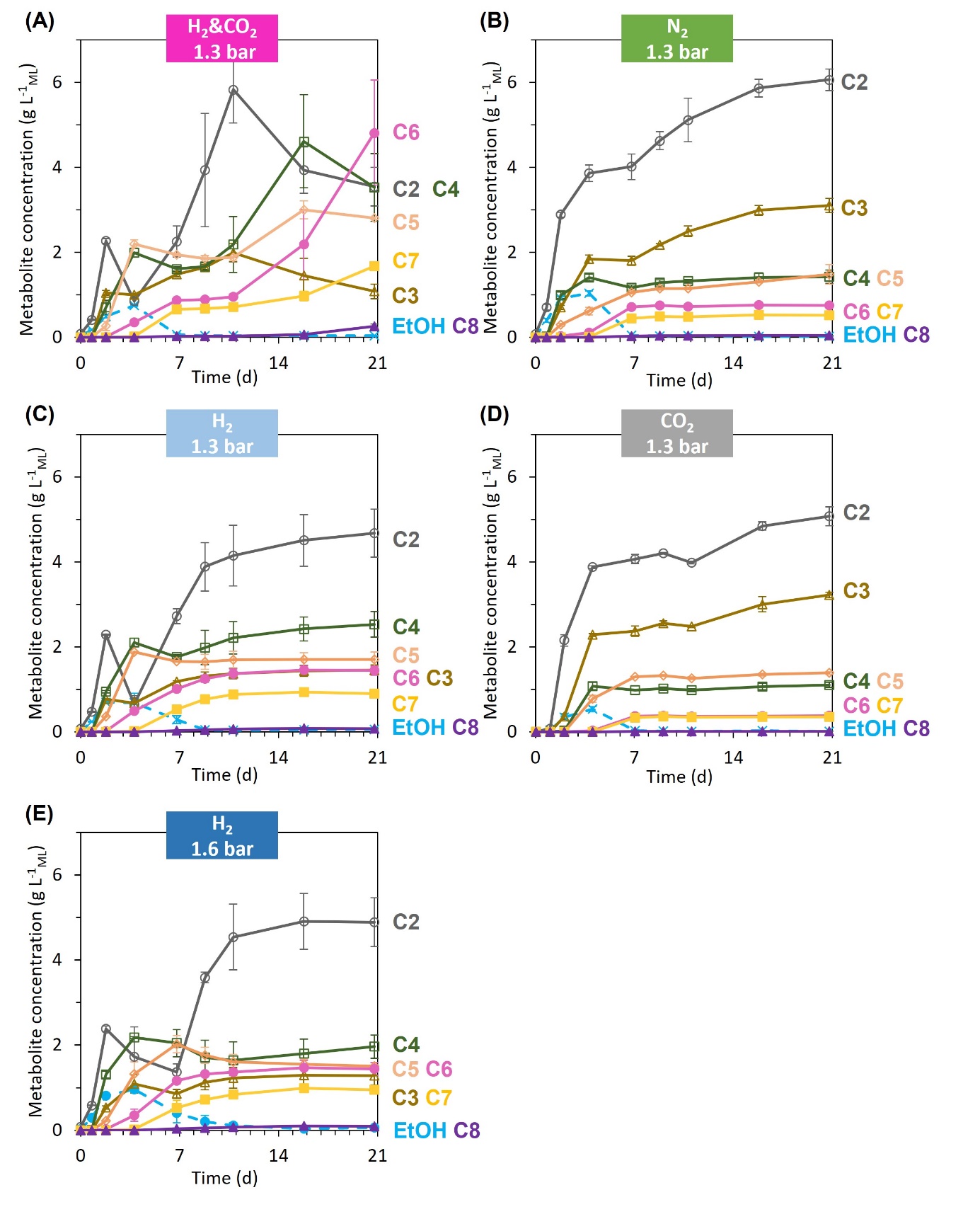


**Fig. S6.** Evolution of the main metabolite concentrations during the 21 days of fermentation under the different conditions tested.


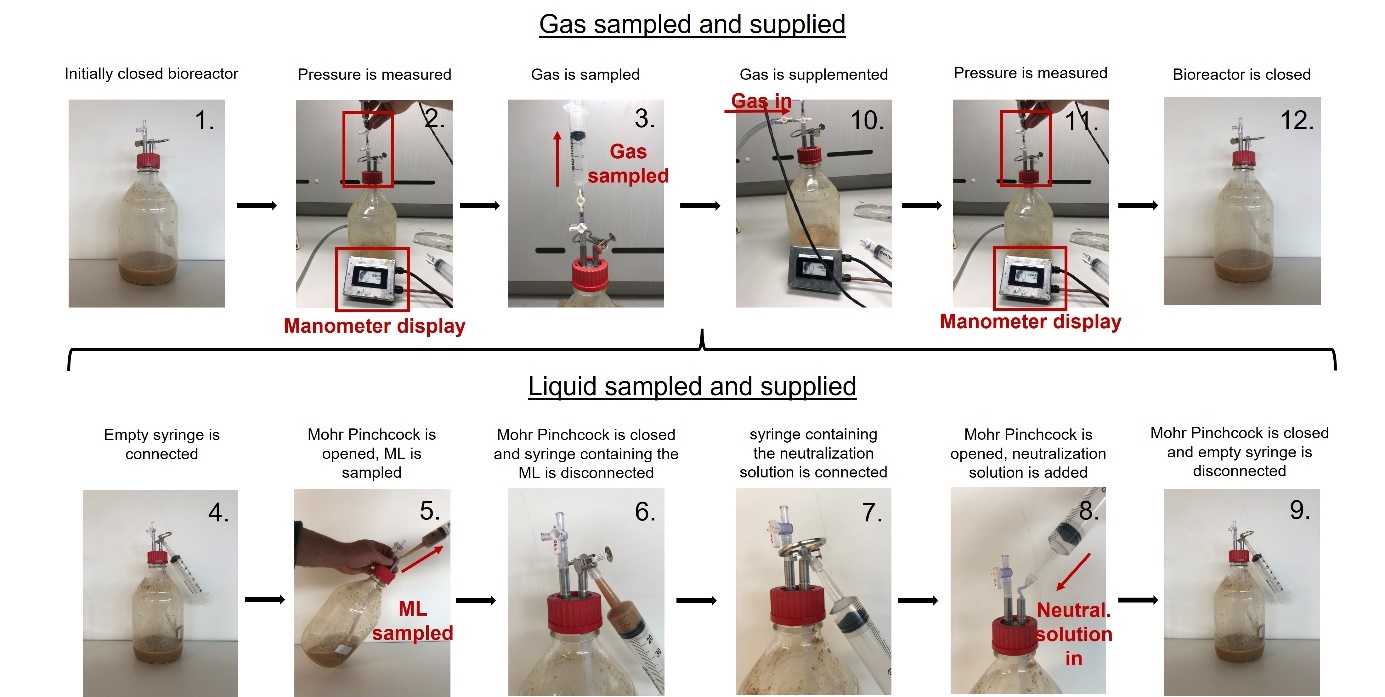


**Fig. S7.** Description of routine monitoring procedure.

# Supplementary Equations

## Theoretical oxygen demand

The theoretical chemical oxygen demand (ThCOD) of a generic organic molecule C_n_H_a_O_b_N_c_ was calculated following **Eq. S1**.

$\text{ThCOD= }\frac{\text{w mol }\text{O}_{\text{2}}}{\text{mol }\text{C}_{\text{n}}\text{H}_{\text{a}}\text{O}_{\text{b}}\text{N}_{\text{c}}}\text{=}\frac{\text{n+}\left( \text{a}/\text{4} \right)\text{-(}\text{b}/{\text{2)-(}\text{3c}/{\text{4}\text{)}}}}{\text{mol }\text{C}_{\text{n}}\text{H}_{\text{a}}\text{O}_{\text{b}}\text{N}_{\text{c}}}\text{= }\left[ \frac{\text{mol}\text{O}_{\text{2}}}{\text{mol}_{\text{molecule}}} \right]\text{ }$ **Eq. S1.**

## Estimated sampling out COD

The value “Estimated sampling solid out” was calculated following **Eq. S2**, with the hypothesis that the solid fraction extracted during the fermentation at each sampling day contained at least the COD of the final solid fraction. As BSG is solubilized during fermentation, the COD of the solid fraction decreased during the fermentation. This calculated value is thus underestimated.

$\text{gCOD}_{\text{Estimated sampling solid out}}\text{= }\sum\text{m}_{\text{Solid fraction sampled}}\text{× }\frac{\text{gCOD}}{\text{g}_{\text{Solid fraction end}}}$ **Eq. S2.**

# References

Hanselmann, K. W. (1991). Microbial energetics applied to waste repositories. *Experientia* 47, 645–687. doi: 10.1007/BF01958816.

Haynes, W. M. (2014). *CRC Handbook of Chemistry and Physics*. CRC Press

Shock, E. L., and Helgeson, H. C. (1990). Calculation of the thermodynamic and transport properties of aqueous species at high pressures and temperatures: Standard partial molal properties of organic species. *Geochim. Cosmochim. Acta* 54, 915–945. doi: 10.1016/0016-7037(90)90429-O.
